# Analysis of the genomic sequences and metabolites of *Serratia surfactantfaciens* sp. nov. YD25^T^ that simultaneously produces prodigiosin and serrawettin W2

**DOI:** 10.1186/s12864-016-3171-7

**Published:** 2016-11-03

**Authors:** Chun Su, Zhaoju Xiang, Yibo Liu, Xinqing Zhao, Yan Sun, Zhi Li, Lijun Li, Fan Chang, Tianjun Chen, Xinrong Wen, Yidan Zhou, Furong Zhao

**Affiliations:** 1 College of Life Sciences, Shaanxi Normal University, Xi’an, 710119 People’s Republic of China; 2 School of Life Sciences and Biotechnology, Shanghai Jiao Tong University, Shanghai, 200000 China; 3 College of Food and Biological Engineering, Jimei University, Xiamen, 361000 China

**Keywords:** *Serratia*, Antimicrobial activity, Genome comparisons, Serrawettin W2, Non-ribosomal peptide synthetases, Quorum sensing, Polyphasic taxonomy

## Abstract

**Background:**

Gram-negative bacteria of the genus *Serratia* are potential producers of many useful secondary metabolites, such as prodigiosin and serrawettins, which have potential applications in environmental bioremediation or in the pharmaceutical industry. Several *Serratia* strains produce prodigiosin and serrawettin W1 as the main bioactive compounds, and the biosynthetic pathways are co-regulated by quorum sensing (QS). In contrast, the *Serratia* strain, which can simultaneously produce prodigiosin and serrawettin W2, has not been reported. This study focused on analyzing the genomic sequence of *Serratia* sp. strain YD25^T^ isolated from rhizosphere soil under continuously planted burley tobacco collected from Yongding, Fujian province, China, which is unique in producing both prodigiosin and serrawettin W2.

**Results:**

A hybrid polyketide synthases (PKS)-non-ribosomal peptide synthetases (NRPS) gene cluster putatively involved in biosynthesis of antimicrobial serrawettin W2 was identified in the genome of YD25^T^, and its biosynthesis pathway was proposed. We found potent antimicrobial activity of serrawettin W2 purified from YD25^T^ against various pathogenic bacteria and fungi as well as antitumor activity against Hela cells. Subsequently, comparative genomic analyses were performed among a total of 133 *Serratia* species. The prodigiosin biosynthesis gene cluster in YD25^T^ belongs to the type I *pig* cluster, which is the main form of *pig*-encoding genes existing in most of the pigmented *Serratia* species. In addition, a complete autoinducer-2 (AI-2) system (including *lux*S, *lsr*BACDEF, *lsr*GK, and *lsr*R) as a conserved bacterial operator is found in the genome of *Serratia* sp. strain YD25^T^. Phylogenetic analysis based on concatenated Lsr and LuxS proteins revealed that YD25^T^ formed an independent branch and was clearly distant from the strains that solely produce either prodigiosin or serrawettin W2. The Fe (III) ion reduction assay confirmed that strain YD25^T^ could produce an AI-2 signal molecule. Phylogenetic analysis using the genomic sequence of YD25^T^ combined with phylogenetic and phenotypic analyses support this strain as a member of a novel and previously uncharacterized *Serratia* species.

**Conclusion:**

Genomic sequence and metabolite analysis of *Serratia surfactantfaciens* YD25^T^ indicate that this strain can be further explored for the production of useful metabolites. Unveiling the genomic sequence of *S. surfactantfaciens* YD25^T^ benefits the usage of this unique strain as a model system for studying the biosynthesis regulation of both prodigiosin and serrawettin W2 by the QS system.

**Electronic supplementary material:**

The online version of this article (doi:10.1186/s12864-016-3171-7) contains supplementary material, which is available to authorized users.

## Background

Gram-negative bacteria of the genus *Serratia* have been isolated from water, air, soil, plants, and animals and are members of the *Enterobacteriaceae* [1]. The ubiquity of *Serratia* is largely attributed to the variety of compounds that are released into the environment [2, 3]. Some species of *Serratia* such as *S. plymuthica*, *S. rubidaea*, *S. marcescens* and *S. nematodiphila* produce a non-diffusible red pigment identified as prodigiosin, which is an alkaloid secondary metabolite with a unique tripyrrole chemical structure [4]. In addition, some species of *Serratia* also produce various useful secondary metabolites including oocydin A, carbapenem, althiomycin, bacteriocins, and serrawettins [5–7]. These useful secondary metabolites have potential applications in the environmental bioremediation and pharmaceutical industry.

Prodigiosin has been shown to have antimicrobial (antifungal, antibacterial, antiprotozoal), antimalarial, antitumor, and immunosuppressant activities at nontoxic levels [8–10]. Other important secondary metabolites are serrawettins, which are useful biosurfactants produced by *Serratia* [11]. Three molecular species, serrawettin W1, W2, and W3, have been reported [12]. Serrawettin W1 is a symmetric dilactone structure composed of two serine residues connected with two 3-hydroxydecanoic acids [13]. It has been regarded as a good anti-cancer drug, which could inhibit cell growth and induce apoptosis of several cell lines derived from T-cell leukemia or Burkitt lymphoma [14, 15]. Serrawettin W2 contains a fatty acid connected with five amino acid residues, which was first isolated from *S. marcescens* in 1986 [16]. Serrawettin W2 is a biosurfactant that can disperse *Caenorhabditis elegans* [17], and antimicrobial activity against *Staphylococcus aureus* has been reported [18]. Moreover, there are fewer reports about the bioactivity of cyclic lipopeptides serrawettin W2 and W3. It was found that several *Serratia* strains, including *S. marcescens* ATCC 274 [19], *S. marcescens* 2170 [20], *S. marcescens* CH-1 [21], and *S. marcescens* NS-38 [12], could produce prodigiosin and serrawettin W1 at the same time. However, the strains that could parallel-produce prodigiosin and serrawettin W2 as the main bioactive compounds have not previously been published.

Many active metabolites produced by *Serratia* strains are regulated by quorum sensing (QS), including butanediol fermentation; production of exoenzymes; nuclease and secondary metabolites such as biosurfactant, carbapenem, oocydin A; and prodigiosin [22–26]. Furthermore, a wide spectrum of important processes, such as bioluminescence, motility, sporulation, virulence, and biofilm formation, are also regulated by the QS system, which influences bacteria community gene regulation by cell–cell communication via the production and detection of diffusible auto-inducer signaling molecules [27]. Generally, the most extensively described QS system in Gram-negative bacteria is the autoinducer-1 (AI-1)-mediated system, which employs N-acyl-L-homoserine lactone (AHL) as the autoinducer signaling molecules [28, 29]. Another type of diffusible autoinducer molecules is autoinducer-2 (AI-2), whose synthesis is dependent on LuxS [30, 31]. In *Enterobacteriaceae*, the AI-2 is internalized into the cells by means of an ABC transporter encoded by *lsr* operon, and intracellular AI-2 is phosphorylated by LsrK. Subsequently, the phosphorylated form of the signal (AI-2-P) binds LsrR, the repressor of the *lsr* operon, and induces *lsr* transcription simultaneously. The further processing of intracellular AI-2-P is then required by LsrF and LsrG proteins [32, 33]. At present, increasingly more AI-2-mediated systems are continuously discovered and explored in *Serratia,* which is also taxonomically classified as *Enterobacteriaceae* [34–36]. Nevertheless, the genetic characteristics and molecular mechanisms about the AI-2 systems of *Serratia* sp. are still less well understood.

In recent years, increasingly more new *Serratia* species have been identified [37]. To date, eighteen species have been known to belong in the genus *Serratia*. The availability of complete bacterial genomes has provided new possibilities for bacterial species classification [38–40]. The most relevant comparative parameter for ascertaining the identity of a strain is the calculation of the average nucleotide identity (ANI), a highly accurate technique that measures the genetic and evolutionary distance between two genomes [41]. OrthoANI, using the recently improved new ANI algorithm, is used as an alternative to DNA–DNA hybridization (DDH) for species delineation, and the 95-96 % OrthoANI value is equivalent to the 70 % DDH threshold that is frequently used for species demarcation [42, 43]. Genome-to-genome distance calculator (GGDC) analysis is another parameter based on genome sequences, which have been proposed to discriminate species [44, 45]. Furthermore, multi-locus sequence analysis (MLSA) has been recommended as a replacement for DDH in species delineation for taxonomic studies [46].

In this work, analysis of genomic sequence and metabolites from a novel pigment-producing strain YD25^T^ isolated from rhizosphere soil was reported. The antagonistic capability of YD25^T^ against vital fungal and bacterial pathogens was explored. The main antibacterial compounds simultaneously produced by YD25^T^ were prodigiosin, serrawettin W2, and seven other putative serrawettin W2 analogues. With the completion of the genome sequence of YD25^T^, taxonomic comparative analysis of the genome and phenotypic analysis data support this strain as a member of a novel and previously uncharacterized *Serratia* species. Additionally, the comprehensive comparative-genomic analysis and mining of the biosynthetic gene cluster and regulation mechanism of secondary metabolites were also performed.

## Methods

### Strain isolation and cultivation

The rhizosphere soils under continuously planted burley tobacco were collected from Yongding, Fujian province, China. The site belongs to a subtropical maritime monsoon climate zone and is characterized by hill-gully. The rhizosphere soil was sandy soil, and the plants were infected by the tobacco mosaic virus and pathogen *Ralstonia solanacearum* due to continuous planting. One gram of the sample was diluted with sterile deionized distilled water serially and spread onto a King’s B medium plate (KB) supplemented with ampicillin (200 mg/mL), chloramphenicol (34 mg/mL) and cycloheximide (80 mg/mL). The agar plates were incubated at 30 °C, and the colonies that appeared were picked up and streaked onto fresh agar plates to obtain pure cultures. In our laboratory, the strain was maintained at −80 °C in 20 % (w/v) glycerol and at 4 °C in culture medium. Morphological features were examined by light microscopy and scanning electron microscopy (S3400, Hitachi) at 30000× magnification.

### Antagonistic bioassays of pathogenic bacteria and fungi

*Exserohilum turcicum*, *Fusarium oxysporum*, *Alternaria alternata* and *Cochliobolus sativus*, all of which are common plant pathogens, were investigated for antagonistic activities of YD25^T^ against fungus. The fungal antagonistic assays were performed following the methods in previous reports [47]. *Micrococcus luteus* and *Ralstonia solanacearum* were used as the indicator microorganisms to investigate the antagonistic activities of YD25^T^ against bacterial pathogens. The biofilm and planktonic culture were used to assay antibacterial competition as described previously [48]. Samples containing only YD25^T^ or bacterial pathogens were set up as controls. Three independent experiments were performed for each assay.

### Extraction, purification and structure identification of the active compounds

YD25^T^ was inoculated in 50 mL of LB medium at 30 °C for 24 h. The seed culture was transferred to 200 mL of KB medium and incubated for another 72 h. The culture was centrifuged to obtain cells, and the cells were washed with deionized H_2_O twice and then extracted with 10 volumes of ethanol acidified with HCl (pH 2.0). The sediments and ethanol were removed by centrifugation and evaporation, respectively. The dry material was then further extracted with an excess of ethyl acetate. The crude extract was further purified by flash chromatography employing 200-300 mesh silica gel, while collected fractions were appraised with thin layer chromatography and UV-vis spectral analysis. Oil displacement activity and antibacterial activity of the collected fractions were also checked at the same time. The solvent used for the purification of the crude was ethyl acetate: petroleum ether (1:1, v/v) followed by ethanol.

The fraction eluted with ethanol was further purified using a semi-preparative HPLC system (Shimadzu LC-8A, Japan) equipped with a Sinochrome ODS BP C_18_ column (10 μm, 20 × 250 mm, Dalian Elite, Dalian, China). Eluent A was composed of purified water containing 0.05 % trifluoroacetic acid, and methanol was selected as eluent B. The following gradient of eluent B was used to run the column: 87-92 % for 0-15 min, 92-97 % for 15-35 min. The flowrate was 10 mL/min. UV detection was performed at 215 nm. The purity of each component was evaluated by an analytical HPLC system (Shimadzu LC-20AT, Japan) equipped with a Sinochrome ODS BP C_18_ column (5 μm, 4.6 × 250 mm, Dalian Elite). The solvent system and the timetable were the same as the semi-preparative HPLC. The flowrate was 0.6 mL/min.

Electrospray ionization (ESI) mass spectra were acquired on a Bruker ion trap mass spectrometer (Esquire 6000, Bruker, Karlsruhe, Germany) coupled with an Agilent 1100 series HPLC. The purified components were infused to the mass spectrometer directly. One component sw-5 was further identified by nuclear magnetic resonance (NMR). Sw-5 was dissolved in 500 μL of dimethyl sulfoxide-d^6^. NMR spectra were then recorded at 600 and 153 MHz (Bruker AM 600) for ^1^H- and ^13^C-NMR, respectively. Complete chemical shift assignments of sw-5 were supported by 2D NMR (HMQC, HMBC), and the amino acid sequencing was determined by HMBC and ROESY experiments.

### Biological activity assays

Production of YD25^T^ antibiotic compounds was tested against bacterial pathogens *Bacillus subtilis* A47 and *M. luteus* CGMCC 1.2299 using the paper disc method. To further examine the antibacterial spectrum of sw-5, an expanded indicator panel composed of Gram-negative and Gram-positive bacteria were tested, including *Escherichia coli* 44102, *Pseudomonas aeruginosa* A62, *Rhodococcus rhodochrous* CGMCC 4.1815, *Enterococcus faecium* CGMCC 1.2025, *Klebsiella pneumoniae* CGMCC 1.10617, *Psychrobacter faecalis* CGMCC 1.10869, *Acinetobacter baumannii* CGMCC 1.6769, *Shigella dysenteriae* CGMCC 1.1869, and the drug-resistant *S. aureus* clinical isolates. *E. coli* 44102, *P. aeruginosa* A62, *B. subtilis* A47 and the clinically relevant pathogens *S. aureus* were purchased from Shaanxi Institute of Microbiology. The other tested strains were purchased from China General Microbiological Culture Collection Center (CGMCC). The culture of indicator strain was diluted to 10^8^ colony forming units (CFU)/mL, and 100 μL of the dilutions was spread on an LB agar plate. A paper disc (6 mm in diameter) impregnated with 10 μL of YD25^T^ compounds (300 μg/mL) dissolved in methanol was placed on the surface of the agar plate. Meanwhile, the paper disc impregnated with 10 μL of methanol was used as a control. The diameter of the inhibition zone was measured after incubation for 24 h.

Cancer cell lines (human cervical cancer cells, HeLa; human epithelial colorectal adenocarcinoma cells, Caco2) and nonmalignant cell lines (human embryonic kidney cell line 293, HEK293; African green monkey kidney cell line, Vero) were incubated with increasing concentrations of sw-5 (3.25-40 μM) to examine the anti-tumor activity of YD25^T^ compounds. After 24 h, 10 μL of MTT solution (Sigma) at 5 mg/mL in PBS was added to each well and incubated for 4 h. The blue MTT formazan precipitate was dissolved in 100 μL of DMSO, and the absorbance at 550 nm was determined.

### Genome sequencing, assembly and annotation

Paired-end (PE) strategies with Illumina technology were performed at life sequencing SL (Valencia, Spain) using next-generation sequencing (NGS) to obtain the almost complete genome sequence of YD25^T^. A strategy using short (approximately 500 nts sequenced by Miseq PE300) and long (consensus sequences of up to 3 kb sequenced by Hiseq2500 PE125) fragments was used to improve the assembly. *De novo* assembly was performed by the sequencing company using Velvet v 1.2.10. Genome annotation was performed using the life sequencing annotation pipeline using Blast. Contigs were assembled using Velvet software (version v 1.2.10). Sequences were annotated using the NCBI Gene Locator and Interpolated Markov ModelER (GLIMMER; http://www.ncbi.nlm.nih.gov/genomes/MICROBES/glimmer_3.cgi) (locus tag VIB2010). BLASTp [49] was applied to align the amino acid sequences against the COG/SwissProt databases [50, 51].

### Genome mining of YD25^T^

To identify the potential NRPS/PKS secondary metabolite biosynthesis gene clusters encoded within the YD25^T^ genome, the bioinformatics tool anti-SMASH was employed [52]. To investigate the likely function of prodigiosin biosynthesis gene clusters, manual analysis using BLASTp was performed. Open reading frames (ORFs) in the serrawettin W2 and prodigiosin gene cluster were automatically predicted using Glimmer 3.0, available on the National Center for Biotechnology Information (NCBI) website [53]. Multiple alignments were performed using the program Clustal W [54]. Phylogenetic analysis was performed by using MEGA version 6.0 [55] and the neighbor-joining method [56] based on the Kimura two-parameter model of evolution [57].

We retrieved all complete genomic sequences of *Serratia* sp. including 133 strains present in Genbank (http://www.ncbi.nlm.nih.gov/genome/, last accessed January 20, 2016). For QS analysis, 43 strains are representatives of bacteria with a complete AI-2 system that were chosen from 133 strains to perform the phylogenetic analysis of AI-2 in *Serratia* sp. The gene content of the *lsr* (*lux*S regulated) clusters was manually identified in each strain. Multiple sequence alignments of the *lsr* clusters were carried out with Clustal W, and then an NJ tree was constructed consisting of ten concatenated genes-*lsr*A, *lsr*B, *lsr*C, *lsr*D, *lsr*E, *lsr*F, *lsr*G, *lsr*K, *lsr*R and *lux*S, based on the Kimura two-parameter model.

### AI-2 bioassay

The detection of AI-2 using the Fe (III) ion reduction assay was performed as previously reported [58]. A working solution of 10 mM 1,10-phenanthroline/3.32 mM Fe (III) was prepared by dissolving 1,10-phenanthroline and ferric ammonium sulfate in deionized distilled water and was adjusted to pH 2 using 1 M HCl. A fresh 1 mg/mL stock solution of ascorbic acid in deionized distilled water was used for standardization and as a positive control for the reduction of Fe (III) ion.

YD25^T^ was grown in KB medium overnight at 30 °C, and then the resulting seed culture was 1/1000 inoculated into the fresh medium and cultured at 30 °C, 200 rpm. Samples were taken from flasks every 2 h over a period of 24 h, and the total number of viable cells was determined by the spreading plate method. At the same time, cell-free supernatant sample (CFS) was prepared by centrifuging cultures at 12,000 rpm for 10 min and passing the medium through 0.22 μm Millipore membrane filters. For detection of AI-2 in CFS, 100 μL of CFS was mixed with 100 μL of Fe (III)-1,10-phenanthroline reagent and left to stand for 1 min to develop the full color. The solution was then diluted to 500 μL using KB medium and scanned for the absorption spectrum against a blank solution within 3 min using a Microplate Luminometer (Epoch, USA). The negative and positive controls used were KB medium without and with ascorbic acid (60 μg/mL), respectively. All assays were performed in triplicate.

### Sequence analyses and genome-wide comparative analysis

For strain identification, genomic DNA was extracted and purified by using a MiniBEST bacterial genomic DNA purification kit (TaKaRa). The primers used for PCR amplification of the 16S rRNA gene were the universal primers: 27 F (5’-AGAGTTTGATCCTGGCTCAG-3’) and 1429R (5’-AAGGAGGTGATCCAAGCCGCA-3’) [59]. The amplified 16S rRNA gene was sequenced by TaKaRa. The calculation of pairwise gene sequence identities used the Web-based EzTaxon-e program (http://eztaxon-e.ezbiocloud.net/) [60]. The ANI calculations were determined in this study using the complete genome sequences of *Serratia* strains available at NCBI and whole-genome sequences from YD25^T^, *S. nematodiphila* DSM 21420^T^, *S. marcescens* LMG 2792^T^, *S. ureilytica* Lr5/4 LG59, *S. proteamaculans* LMG 8751^T^, *S. liquefaciens* LMG 7884^T^, *S. plymuthica* S13, *S. symbiotica* DSM 23270^T^, *S. odorifera* LMG 7885^T^, *S. fonticola* LMG 7882^T^. The ten genomes were uploaded into the software package (http://www.ezbiocloud.net/sw/oat) to perform pairwise genome calculations of OrthoANI using the recently improved new ANI algorithm [61]. DNA-DNA hybridization values between strain YD25^T^ and strains of phylogenetically related species were calculated using the GGDC available online (http://ggdc.dsmz.de/distcalc2.php) with the formula 2 method. In addition, the *rpo*B, *gyr*B, *inf*B and *atp*D genes were amplified and sequenced. The sequences of type strains were available from the Institut Pasteur multi-locus sequence typing (MLST) Web site (www.pasteur.fr/mlst). Each gene was blasted against the nucleotide collection (nr/nt) using the BlastN algorithm (http://blast.ncbi.nlm.nih.gov/).

### Phenotypic and fatty acid analyses

The physiological and biochemical characteristics were examined according to standard methods described by Grimont et al*.* [62]. The strain was characterized phenotypically using BiOLOG GEN III microplate (Biolog) and VITEK 2 GN microplate (bioMérieux VITEK-2 Compact) identification systems. Antibiotic sensitivity tests were performed on KB agar at 30 °C containing the following antibiotics: streptomycin, kanamycin, ampicillin, chloramphenicol, tetracycline, carbenicillin, gentamicin, and apramycin. For quantitative analysis of the cellular fatty acid composition, a loopful of cell mass was harvested, and whole-cell fatty acids were analyzed by fatty acid methyl esters (FAMEs).

## Results

### Antagonistic effects of YD25^T^ on pathogenic bacterial and fungal strains

Strain YD25^T^ was isolated from rhizosphere soils under continuously planted burley tobacco in Fujian Province, China. YD25^T^ produced pigments at 30 °C, which resulted in red colonies on KB medium but could not produce pigments at 37 °C. When it was point-inoculated onto the surface of a KB plate for 24 h, circular irregular margin morphology was observed. Cells are motile, non-spore-forming short rods, 1.2-1.4 × 0.6-0.7 μm and with one weak flagellum (Additional file 1: Figure S1).

**Fig. 1:**
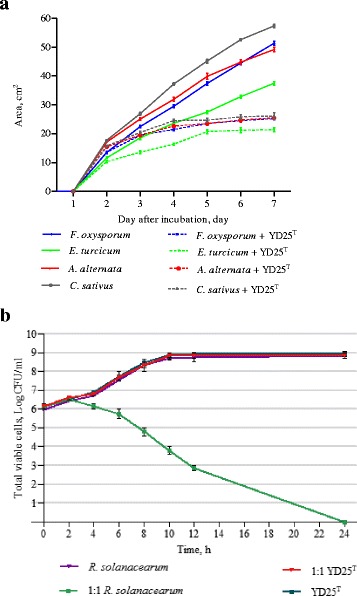
Antagonistic activities of YD25^T^ against plant pathogenic fungi and *Ralstonia solanacearum*. **a** Growth of *E. turcicum*, *F. oxysporum*, *A. alternate* and *C. sativus* with and without YD25^T^ were closely monitored. **b** The planktonic culture assay of the quantities of *R. solanacearum* and YD25^T^ recorded at 0, 2, 4, 6, 8, 10, 12 and 24 h after incubation. Numbers show an average of three replications, and error bars show standard errors of the means

In the bacterial–fungal confrontation assays, all tested fungi were inhibited by the YD25^T^ colony as early as the second day and completely inhibited on the fourth day of inoculation (Fig. 1a). All fungi that did not grow past the YD25^T^ colony streaked across the agar plates, though the fungal mycelia also turned a darker color at the later stages of bacteria–fungi interaction (Additional file 2: Figure S2A). The noncontact inhibition seemed to be more evident in *C. sativus* than others.

The biofilm culture assay showed that *M. luteus* and *R. solanacearum* were dramatically inhibited when they were co-cultured with YD25^T^ at an initial cell ratio of 1:1 after an 8-h incubation. A comparison of the culture without and with YD25^T^ revealed a 10^4^ times increase of *M. luteus* and 10^5^ times increase of *R. solanacearum* in the number of viable cells recovered (Additional file 2: Figure S2BC). The planktonic culture assay showed a similar result when YD25^T^ and *R. solanacearum* were co-cultured at an initial ratio of 1:1 (Fig. 1b). Based on these experiments, it was concluded that YD25^T^ was capable of inhibiting the growth of plant fungal pathogens and bacterial pathogens.

### Purification and structure analysis of the active compounds of YD25^T^

**Fig. 2:**
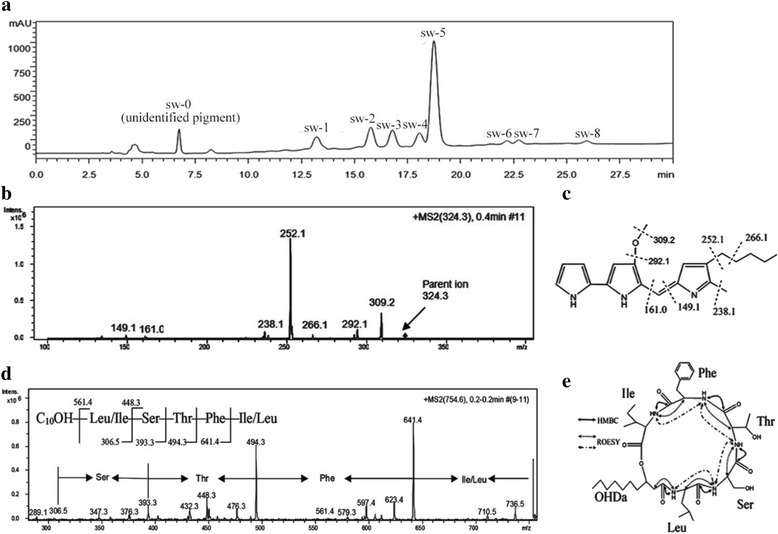
HPLC analysis and structure analyses of the main compounds produced by YD25^T^. **a** HPLC analysis of the fraction extracted from YD25^T^; **b** The ESI-MS/MS analyses of sw-0 extracted from YD25^T^; **c** The structure of sw-0 has been constructed with the positions of bonds whose breakage resulted in the formation of the respective fragments; **d** The ESI-MS/MS analyses of component sw-5; **e** The structure of sw-5 has been constructed, and key ROESY and HMBC correlations for sw-5 are shown

Nine compounds named sw-0 to sw-8 were identified (Fig. 2a). The isolated sw-0 had the maximum absorption spectrum at 532 nm under acidic conditions and at 470 nm under alkaline conditions. ESI–MS analysis indicated that sw-0 had a molecular mass of 323.2 Da. Induced dissociation of the “parent” ion (324.3 Da) revealed the following fragments of the molecular ion: 149.1, 161.0, 238.1, 252.1, 266.1, 292.1 and 309.2 Da (Fig. 2b). The molecular weight of the “parent” ion of sw-0 and the weights of the fragments exactly corresponded to the prodigiosin with elemental composition of C_20_H_25_N_3_O (Fig. 2c).

Sw-5 was the most abundant compound. Based on ion peaks at m/z 732.2 [M + H]^+^, m/z 754.6 [M + Na]^+^, and m/z 770.6 [M + K]^+^, the molecular mass of sw-5 was determined to be 731.2 Da. Some amino acid residues and their connecting relationship could be determined by comparison of the differences between peaks in MS spectra. Thus, the [M + Na]^+^ ion was chosen as the precursor ion for further collision-induced dissociation analysis. As shown in the MS/MS spectrum (Fig. 2d), sets of fragment ions were observed. Because Leu and Ile shared the same molecular weight, peaks 754.6 → 641.4 → 494.3 → 306.5 suggested the connection of amino acid residues in the form of Leu-Phe-Thr-Ser or Ile-Phe-Thr-Ser. Peak 561.4 indicated an Ile/Leu linked to the fatty acid chain. Another series fragment formed as an ester bond was cleaved following the double hydrogen transfer mechanism [20]. The peaks of 376.3, 476.3, 579.3 and 623.4 were due to the losses of Thr-Phe-Leu/Ile-H_2_O (378.3 Da), Phe-Leu/Ile-H_2_O (278.3 Da), C_10_H_14_-Na-H_2_O (175.2 Da) and Leu/Ile-H_2_O (131.2 Da). These fragments gave the same structure information in accordance with the above analysis. To further confirm the structure of sw-5, NMR was carried out to identify the amino sequence and the positions of linkages within the carbohydrate and lipid molecules. The complete ^13^C NMR chemical shift and partly ^1^H chemical shift assignments are shown in Additional file 3: Table S1. The NMR results demonstrated that the fatty acid residue was β-hydroxydecanoic acid, and five different amino acids constituted the peptide chain. In addition, the chemical shift (δ_c_, 71.7; δ_H_, 4.92) of the third position of fatty acid was consistent with the shift due to lactone ring formation. The sequence of the residues of amino acids was Leu-Ser-Thr-Phe-Ile, determined by interpretation of HMBC data, which was corroborated by NOESY evidence (Fig. 2e). According to comparison with literature data [21, 22], sw-5 was identical to serrawettin W2.

The molecular weights of sw-1, sw-2, sw-6, sw-7, and sw-8 are 703.3, 717.3, 745.4, 757.2 and 759.3 Da, respectively. Sw-3 and sw-4 have the same molecular weight (731 Da) as sw-5, although their retention times are 16.809 and 18.101, respectively. The similar analyses of MS/MS spectra were carried out for the putative serrawettin W2 analogues (Table 1). The results indicated that sw-1 and sw-2 varied at the first or fifth amino acid positions, while sw-6, sw-7 and sw-8 varied at the first, second or fifth amino acid positions. For sw-3 and sw-4, different structures of oligopeptides or the fatty acid chain from serrawettin W2 were observed.

**Table 1:** The characteristics and the putative structures of the putative serrawettin W2 analogues

Compound	[M + H]^+^ (m/z)	[M + Na]^+^ (m/z)	[M + K]^+^ (m/z)	MW^b^ (m/z)	MS fragment (ESI-MS/MS)	Structure predicating^c^
sw-1	704.3	726.5	742.4	703.3	613.3,595.3^a^,551.4,533.3^a^,466.3,448.3^a^,	C_10_OH-Abu/Aib-Ser-Thr-Phe-Ile/Leu
					365.2, 347.3^a^	
sw-2	718.3	740.6	756.5	717.3	641.3,627.4^a^,565.3,547.3^a^,494.3,480.3^a^,	C_10_OH-Val-Ser-Thr-Phe-Ile/Leu
					448.3, 379.3, 361.2^a^	
sw-3	732.2	754.6	770.4	731.2	641.4,623.5^a^,579.8,551.4^a^,494.3,476.3^a^,	C_10_OH-Leu/Ile-Ser-Thr-Phe-Ile/Leu
					448.4, 393.3, 375.1^a^	
sw-4	732.3	754.6	770.6	731.3	641.3,623.4^a^,579.3,551.1^a^,494.3,476.3^a^,	C_10_OH-Leu/Ile-Ser-Thr-Phe-Ile/Leu
					448.3, 393.1, 375.2^a^	
sw-6	746.4	768.6	784.5	745.4	655.4,637.2^a^,593.4,575.2^a^,508.3,490.3^a^,	C_10_OH-Leu/Ile-Thr-Thr-Phe-Ile/Leu
					462.3, 407.2, 389.1^a^	
sw-7	758.2	780.5	796.5	757.2	667.4, 649.5^a^, 605.7, 587.4^a^, 520.2,	C_10_OH-Leu/Ile-Leu/Ile-Thr-Phe-Ile/Leu
					502.3^a^, 474.3, 419.2, 402.1^a^	
sw-8	760.3	782.5	798.3	759.3	669.3, 651.2^a^, 625.5, 607.4^a^, 522.3,	C_10_OH-Leu/Ile-Asp-Thr-Phe-Ile/Leu
					504.5^a^, 476.5, 421.3, 403.3^a^	

^a^These peaks were formed as an ester bond was cleaved following double hydrogen transfer (DHT) mechanism. ^b^The molecular weight (MW) was based on the peaks of [M + H]^+^, [M + Na]^+^ and [M + K]^+^. ^c^Mass spectrometry couldn’t distinguish between isomers like leucine and isoleucine

### Biological activities of the active compounds of YD25^T^

**Fig. 3:**
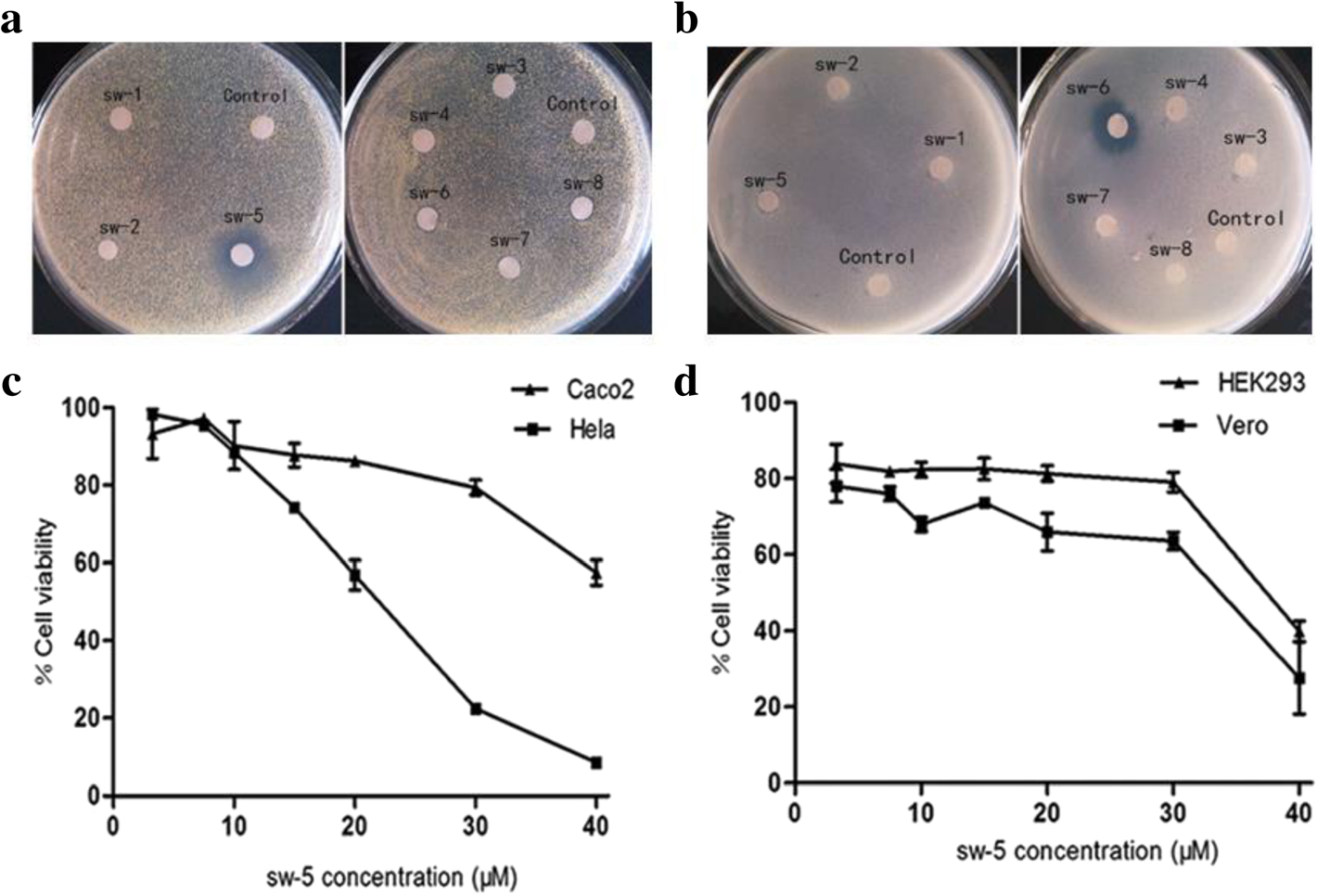
The biological activities of the compounds from YD25^T^. **a** The antibacterial activities of the components against *M. luteus* CGMCC 1.2299; **b** The antibacterial activities of the components against *B. subtilis* A47; **c** The cytotoxicity on Hela and Caco2 cells of sw-5 (serrawettin W2); **d** The cytotoxicity on nonmalignant cells of sw-5 (serrawettin W2). Numbers show an average of three replications, and error bars show standard errors of the means

Among the eight compounds (sw-1, 2, 3, 4, 5, 6, 7, 8) of YD25^T^, sw-5 (serrawettin W2) inhibited *M. luteus* CGMCC 1.2299 growth highly and *B. subtilis* A47 minimally, but sw-6 showed the opposite results. Sw-7 and sw-8 had a slight inhibition to *M. luteus* CGMCC 1.2299 and no activity to *B. subtilis* A47. The other compounds had no antibacterial activities (Fig. 3a, b). To further examine the antibacterial spectrum of serrawettin W2, an expanded indicator panel composed of Gram-negative and Gram-positive bacteria was tested. The result indicated that serrawettin W2 had activities to *R. rhodochrous* CGMCC 4.1815, *S. dysenteriae* CGMCC 1.1869, and *P. aeruginosa* A62. Furthermore, it was noteworthy that among thirty drug-resistant *S. aureus* clinical isolates, serrawettin W2 exhibited evident inhibitory activities to nine strains and slight inhibition to twelve strains (Table 2).

**Table 2:** Antibacterial activity of sw-5 (serrawettin W2) produced by YD25^T^

Bacteria	Drug resistance									Inhibitory zone
	1	2	3	4	5	6	7	8	9	diameter (mm)
*Staphylococcus aureu* A1	R	R	R	R	R	R	R	-	-	NI
*Staphylococcus aureu* A2	R	S	S	R	R	R	R	-	-	SI
*Staphylococcus aureu* A3	S	R	S	S	R	S	S	-	-	NI
*Staphylococcus aureu* A4	R	R	R	R	R	R	R	-	-	NI
*Staphylococcus aureu* A5	R	R	R	R	R	R	R	-	-	SI
*Staphylococcus aureu* A6	R	R	R	R	R	R	R	-	-	SI
*Staphylococcus aureu* B1	S	R	S	R	R	S	S	-	-	NI
*Staphylococcus aureu* B2	R	R	R	R	R	R	R	-	-	SI
*Staphylococcus aureu* B3	R	R	R	R	R	R	R	-	-	NI
*Staphylococcus aureu* B4	S	R	S	S	R	S	S	-	-	NI
*Staphylococcus aureu* B5	S	S	S	S	R	S	S	-	-	SI
*Staphylococcus aureu* B6	S	S	S	S	S	S	S	-	-	SI
*Staphylococcus aureu* C1	R	R	R	R	R	R	R	-	-	NI
*Staphylococcus aureu* C2	R	R	R	R	R	R	R	-	-	SI
*Staphylococcus aureu* C3	S	R	S	S	R	I	S	-	-	11.2 ± 0.4
*Staphylococcus aureu* C4	R	R	R	R	R	R	R	-	-	SI
*Staphylococcus aureu* C5	R	R	R	R	R	R	R	-	-	SI
*Staphylococcus aureu* C6	S	R	S	S	R	S	S	-	-	9.2 ± 0.3
*Staphylococcus aureu* D1	R	S	R	R	R	R	R	-	-	NI
*Staphylococcus aureu* D2	S	R	S	S	R	S	S	-	-	7.6 ± 0.4
*Staphylococcus aureu* D3	R	R	R	R	R	R	R	-	-	SI
*Staphylococcus aureu* D4	R	R	R	R	R	R	R	-	-	NI
*Staphylococcus aureu* D5	R	R	S	R	R	R	R	-	-	8.5 ± 0.5
*Staphylococcus aureu* D6	R	S	S	R	R	R	R	-	-	SI
*Staphylococcus aureu* E1	S	R	S	R	R	S	S	-	-	11.2 ± 0.3
*Staphylococcus aureu* E2	R	S	R	R	R	R	R	-	-	SI
*Staphylococcus aureu* E3	-	-	-	S	-	S	S	R	R	8.3 ± 0.2
*Staphylococcus aureu* E4	-	-	-	-	-	S	S	R	R	11.4 ± 0.4
*Staphylococcus aureu* E5	S	I	S	S	R	S	S	-	-	10.1 ± 0.3
*Staphylococcus aureu* E6	S	R	S	S	R	S	S	-	-	8.1 ± 0.4
*Escherichia coli* 44102	-	-	-	-	-	-	-	-	-	NI
*Pseudomonas aeruginosa* A62	-	-	-	-	-	-	-	-	-	14.4 ± 0.4
*Bacillus subtilis* A47	-	-	-	-	-	-	-	-	-	SI
*Rhodococcus rhodochrous*	-	-	-	-	-	-	-	-	-	12.5 ± 0.3
*CGMCC 4.1815*										
*Enterococcus faecium*	-	-	-	-	-	-	-	-	-	NI
CGMCC 1.2025										
*Klebsiella pneumoniae*	-	-	-	-	-	-	-	-	-	NI
CGMCC 1.10617										
*Psychrobacter faecalis*	-	-	-	-	-	-	-	-	-	NI
CGMCC 1.10869										
*Acinetobacter baumannii*	-	-	-	-	-	-	-	-	-	NI
CGMCC 1.6769										
*Shigella dysenteriae*	-	-	-	-	-	-	-	-	-	10.5 ± 0.5
CGMCC 1.1869										
*Micrococcus luteus*	-	-	-	-	-	-	-	-	-	19.4 ± 0.5
CGMCC 1.2299										

1, prostaphlin (10 units); 2, clindamycin (2 μg); 3, rifampicin (5 μg); 4, levofloxacin (5 μg); 5, erythromycin (15 μg); 6, amikacin (30 μg); 7, cefazolin (30 μg); 8, paediatric compound sulfamethoxazole tablets (23.75 μg); 9, ampicillin (10 μg). R, resistance to the antibiotic; S, sensitive to the antibiotic; I, intermediate; “-”, not determined. NI, no inhibition; SI, slight inhibition. SI indicated the diameter of the inhibition zone was less than 7 mm. The diameter of the control was 6 mm. Numbers show an average of three replications

In addition to the excellent antibacterial activity, serrawettin W2 also exhibited anticancer activity. Serrawettin W2 showed cytotoxicity on Hela and Caco2 cells at concentrations from 3.25 μM to 30 μM. The IC_50_ of serrawettin W2 was 20.9 μM for the Hela cell line and 54.1 μM for Caco2 (Fig. 3c). Thus, serrawettin W2 inhibited growth of Hela cells more significantly than Caco2 cells. For the normal cell lines, serrawettin W2 did not show a marked decrease in viability of Vero cells and HEK293 cells at concentrations of up to 30 μM. Furthermore, a higher concentration (40 μM) of serrawettin W2 resulted in an approximate 60 % cell death (Fig. 3d). These results indicate that serrawettin W2 can significantly suppress the growth of cancer cell lines and has slight effects on the viability of nonmalignant cells.

### Analysis of the genomic sequence to identify putative serrawettin W2 gene cluster

The new genomic sequence for YD25^T^ had 17 contigs with a total length of approximately 5,115,690 bp (Additional file 4: Figure S3). The mean contig size was 300,923 bp, with the N50 (50 % of the genome is contained in contigs of size N or greater) contig size being 1,200,118 bp. Analysis of the genome sequence of YD25^T^ resulted in the identification of a putative cyclic lipopeptide antibiotics serrawettin W2 biosynthesis gene cluster, which is a more than 21 kb interrupted DNA sequence characterized by a hybrid PKS-NRPS system. The PKS gene consists of an acyltransferase (AT), ketosynthase (KS), and a keto reductase (KR) domain. In the putative typical features of the NRPS gene, there are a total of five modules, and each module consists of specific condensation (C), adenylation (A), and thiolation (T) domains. The five modules harbor one thioesterase (TE) domain. The distance between the PKS and NRPS gene is 9478 bp on a linear map of the genome sequence of YD25^T^.

**Fig. 4:**
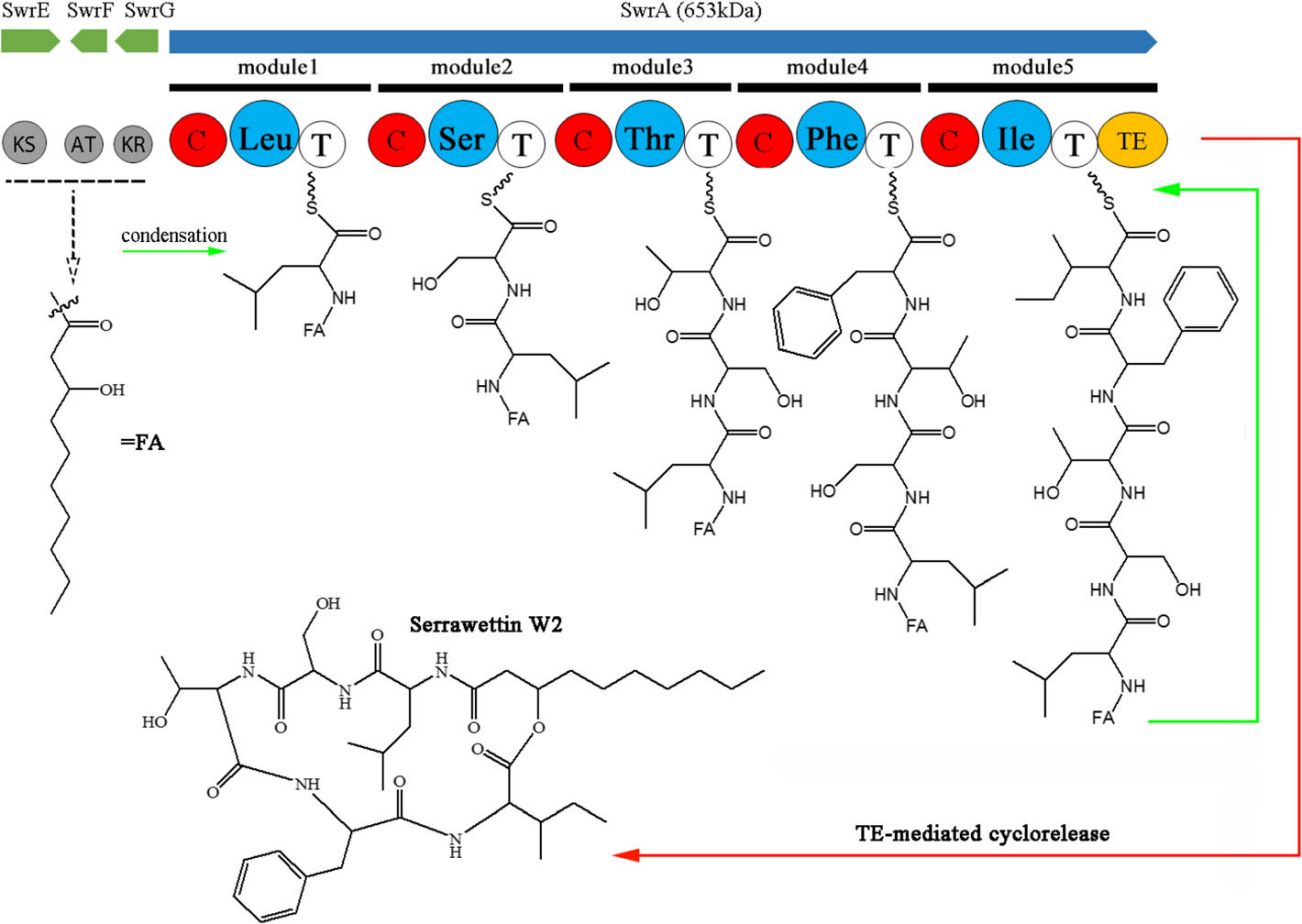
Genetic organization of *swr* biosynthetic gene cluster and proposed biosynthetic pathway for serrawettin W2 identified in YD25^T^. Model for the biosynthesis of serrawettin W2 showing the structures of some of the proposed intermediates. SwrEFG, and SwrA form a hybrid PKS-NRPS system, which consists of five modules as indicated. Underneath the genes are the relative size, module and domain organization, and their predicted products of the PKS-NRPS. The amino acids are predicted to be incorporated into serrawettin W2 peptide moiety based on specific signature sequences in each A-domain. Domains within the PKS-NRPS are as follows. AT: acyltransferase; KS, ketosynthase; KR: ketoreductase; A: adenylation; C: condensation; T: thiolation; TE: thioesterase

Speculation of the domain functions is based on the sequence homology to known PKS and NRPS domains. The putative peptide sequence of serrawettin W2 is Leu-Ser-Thr-Phe/Val-Ile/Val. According to the results of structural analysis of serrawettin W2, the fourth and fifth amino acids are Phe and Ile, and the prediction is in full agreement with the peptide sequence of serrawettin W2 determined by the results of MS. Based on the presence of PKS and NRPS encoding genes, the pathway for serrawettin W2 biosynthesis is deduced (Fig. 4). Firstly, the fatty acid (FA) of the C_10_ unit, which is used as the precursor material, is synthesized by PKS SwrEFG and other undetermined proteins and is released as a fatty acyl-CoA. Secondly, a 17.7 kb *swr*A encodes the core W2-peptide chain (the structure of the CLP with a 5-amino acid peptide moiety), which contains a total of five modules. The N-terminal C-domain of the SwrA initiation module is predicted to catalyze the condensation of the fatty acyl-CoA and leucine. After initiation, chain elongation is mediated by the other three linear domains correlated with the serine, threonine, and phenylalanine, respectively. Finally, the oligopeptide is transferred onto the active-site isoleucine of the C-terminal TE domain of the last module. This organization is consistent with a role in catalyzing the last step of chain elongation, lactonization, and the subsequent cyclorelease of the cyclic lipopeptide. The genome-based identification and structure prediction of serrawettin W2 provides a basis for further improving the production of serrawettin W2 as a useful metabolite by YD25^T^.

### Identification of the prodigiosin biosynthetic gene cluster

**Fig. 5:**
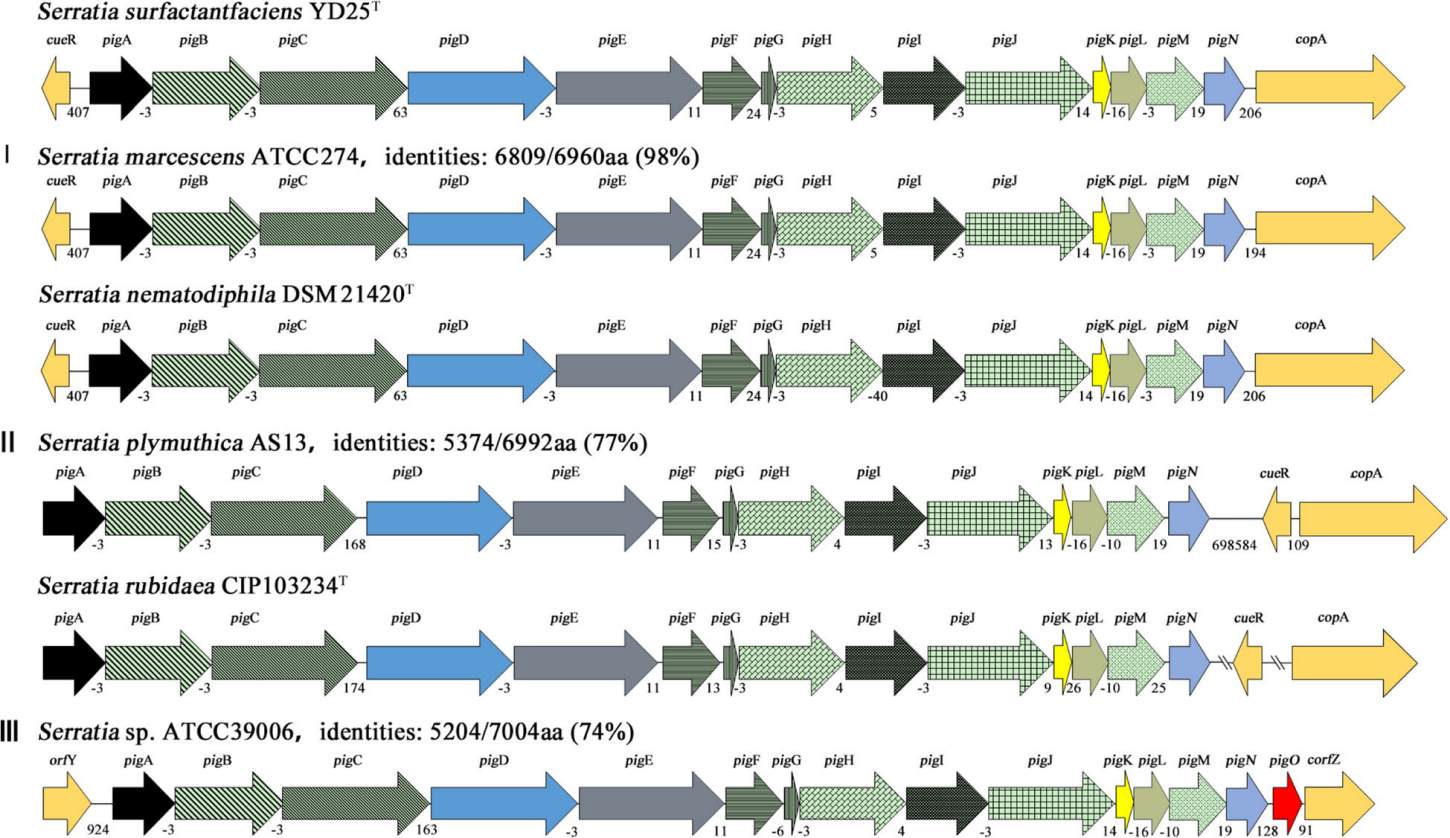
Comparison of prodigiosin biosynthetic gene clusters among pigmented *Serratia* sp. The applicable gene sequence data are from a variety of sources: *S. marcescens* ATCC274, *S. nematodiphila* DSM 21420^T^, *S. plymuthica* AS13, *S. rubidaea* CIP101234^T^, *Serratia* sp. ATCC 39006. The genetic organizations of three types of prodigiosin biosynthetic gene clusters are symbolized by arrows. The positions and intergenic regions of each *pig* gene along a linear representation have been indicated. The identities of the homologue gene clusters between YD25^T^ and the three types are 98 %, 77 %, and 74 %, respectively

A putative prodigiosin gene cluster was found in the genomic sequence of YD25^T^, and putative functions of pig protein were assigned based on the results of BLASTp searches and compared with the other *Serratia* pig proteins in *S. marcescens* ATCC 274, *S. plymuthica* AS 13, and *Serratia* sp. ATCC 39006 (Additional file 5: Table S2). Subsequently, comparative sequence analysis of the *pig* clusters among the pigmented *Serratia* species was shown (Fig. 5). The *pig* clusters were observed in the complete genomic sequences of 34 strains among the total of 133 *Serratia* species present in Genbank (last accessed January 20, 2016). They could be divided into three types: type I-*pig* cluster is flanked by *cue*R and *cop*A in 29 strains represented by *S. marcescens* ATCC 274; type II-*cue*R and *cop*A are adjacent to each other and in the downstream of the *pig* cluster in 4 strains including *S. plymuthica* AS9, *S. plymuthica* AS12, *S. plymuthica* AS13 and *S. rubidaea* CIP103234; type III-*pig*O is present immediately downstream of the *pig* cluster only in *Serratia* sp. ATCC39006. Furthermore, *pig* cluster genes of YD25^T^ showed 98, 77 and 74 % identity at the amino acid level to the three types of *Serratia pig*-encoding genes, respectively. Consequently, the prodigiosin biosynthesis gene cluster in YD25^T^ belongs to the type I *pig* cluster, which is the main form of *pig*-encoding genes existing in the most of pigmented *Serratia* species.

### Analysis of quorum sensing (QS) system and detection of AI-2 signal molecule

**Fig. 6:**
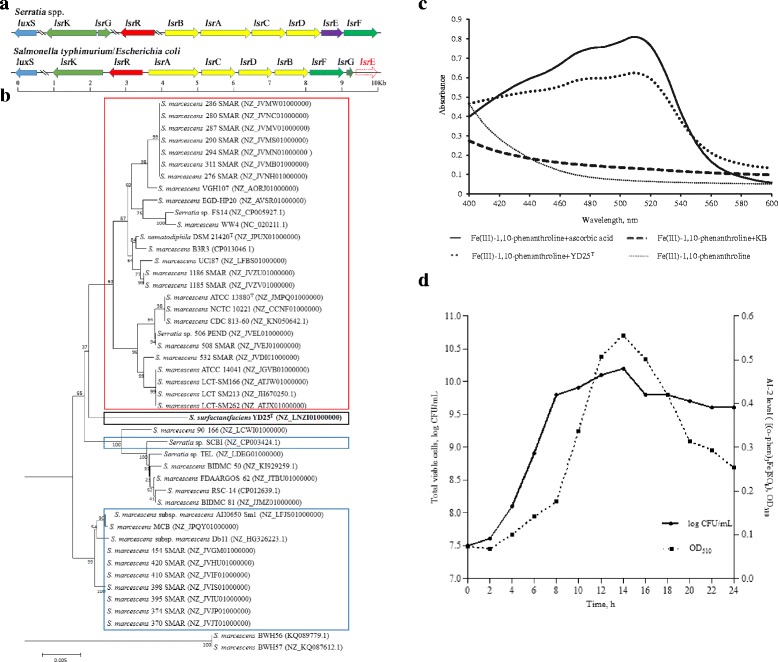
AI-2-mediated quorum sensing system in YD25^T^. **a** Distribution and comparison of gene organization in the complete *lsr* operon between the *Serratia* sp. and the *S. typhimurium*/*E. coli*. The orientation of the arrows indicates the direction of transcription; **b** Phylogenetic relationships based on the concatenated amino acid sequences of complete Lsr protein and LuxS. Boxes represent the strains in which the biosynthetic gene cluster of prodigiosin (red) or serrawettin W2 (blue) are observed in the genome. Bootstrap analysis (1000 resamplings) was used to evaluate the topology of the NJ tree. Bar, 0.005 substitutions per nucleotide position. All sequences were retrieved from the NCBI database or in published genome projects. **c** AI-2 bioassay of YD25^T^ by the Fe (III) ion reduction assay. Representative absorption spectra of 10 mM Fe(III)-1,10-phenanthroline with 60 μg/mL ascorbic acid, a cell-free supernatant sample of YD25^T^ or KB medium; **d** Cell growth of YD25^T^ cultures in KB and the production of extracellular AI-2 levels in the culture media. Numbers show an average of three replications and are derived from one independent sample

AI-1 and AI-2 are the most extensively described QS system in Gram-negative bacteria. For the AI-1 system, two *lux*R homologue genes-*sfc*R and *sfs*R, both with the size of 771 bps-were identified within the YD25^T^ genome. However, no *lux*I homologue gene was identified within the upstream and downstream of *sfc*R and *sfs*R, so we hypothesized that these are solo *lux*R. For the AI-2 system, there are *lux*S and a complete *lsr* gene clusters including *lsr*BACDEF, *lsr*GK, and *lsr*R within the YD25^T^ genome (Fig. 6a). The predicted functions were assigned (Additional file 6: Table S3), and the result of comparison with the *lsr* cluster in *Salmonella typhimurium* LT2, *E. coli* K-12, and *E. fergusonii* ATCC 35469 was shown that the ten proteins in YD25^T^ were similar in size to the homologues encoded in the *lsr* cluster. LuxS and LsrGK exhibited a high degree of sequence similarity at the amino acid level, though LsrBACDEF and LsrR show a lower identity to their homologues from *S. typhimurium* LT2, *E. coli* K-12, and *E. fergusonii* ATCC 35469. To search for orthologs of LuxS and Lsr proteins in *Serratia* sp., we carried out an analysis against all 133 completely sequenced genomes present in the NCBI database as of January 2016. The isolates with Lsr proteins (LsrBACDEF and LsrGK) identified as being orthologs total 80 strains, in which *lsr*R was not detected in the genome sequences of 37 organisms. Therefore, there are 43 strains whose genome sequences include *lux*S and the complete *lsr* operon (*lsr*BACDEF, *lsr*GK, and *lsr*R). Subsequently, the result of phylogenetic analysis using concatenated amino acid sequences of 10 proteins (LuxS, LsrBACDEF, LsrGK and LsrR) among the 43 strains indicated that YD25^T^ formed an independent branch from the other organisms (Fig. 6b). Furthermore, analyzing the gene clusters of prodigiosin and serrawettin W2 within these 43 organisms clearly revealed two distinct groups (group I and group II). Group I includes *Serratia* strains containing only the prodigiosin gene cluster, while group II includes *Serratia* strains containing only the serrawettin W2 gene cluster. Importantly, YD25^T^ producing prodigiosin and serrawettin W2 simultaneously formed an independent branch and was clearly distant from the two groups. These results are the same as the phylogenetic analysis results using the ten-gene (*lsr* operon and *lux*S) sequences (Additional file 7: Figure S4).

In order to examine whether an AI-2 signal molecule is indeed produced in YD25^T^, the Fe (III) ion reduction assay was used as a bioassay method to identify the diffusible autoinducer signaling molecules and detect the level in different bacterial growth phases. In the Fe (III) ion reduction assay, the spectra following the addition of ascorbic acid (as the positive control) or AI-2 to the Fe(III)-1,10-phenanthroline had the maximum absorption wavelength at 510 nm due to the formation of the [(o-phen)_3_Fe]SO_4_ ferroin complex. The AI-2 production of YD25^T^ is shown in Fig. 6c. Representative absorption spectra of 10 mM Fe (III)-1, 10-phenanthroline in the presence of 60 μg/mL ascorbic acid or CFS of YD25^T^ were similar. In contrast, the spectrum of Fe(III)-1,10-phenanthroline alone or with KB medium showed no maximum absorption wavelength at 510 nm. The assay of the AI-2 level in different bacterial growth phases is shown in Fig. 6d. These results clearly indicate that AI-2 was maximally produced by YD25^T^ during the mid-to late-log phase and achieved the maximum at the stationary phase, and then the extracellular AI-2 gradually decreased.

### Taxonomic comparative analysis of the genome of YD25^T^ with other similar species

The resulting 16S rRNA gene sequence (1534 bp) of YD25^T^ was initially deposited in the GenBank database with the accession number KM093865. Comparisons of 16S rRNA gene sequences revealed that the YD25^T^ strain was related most closely to *S. nematodiphila* DSM 21420^T^ (99.7 % similarity) found in *Heterorhabditidoides chongmingensis*. It was also closely related to *S. marcescens* LMG 2792^T^ (99.4 % similarity). Strain YD25^T^ shared similarity less than 98.7 % with other type strains (Additional file 8: Table S4). Phylogenetic analysis based on the neighbor-joining algorithms revealed that strain YD25^T^ was included in the cluster of species of the genus *Serratia*. The phylogenetic tree showed that strain YD25^T^ was related most closely to *S. nematodiphila* and *S. marcescens*. However, the sequence divergence between strain YD25^T^ and the type strains of phylogenetically related species suggested that strain YD25^T^ represents a novel species within the genus *Serratia* (Additional file 9: Figure S5). The mean DNA G + C content of strain YD25^T^ was calculated to be 59.62 mol%. This value is similar to *S. marcescens* LMG 2792^T^ (57.5-60.0 mol%) [2], *S. nematodiphila* DSM 21420^T^ (59.52 mol%) [63], and *S. ureilytica* LMG 22860^T^ (60 mol%) [64].

**Fig. 7:**
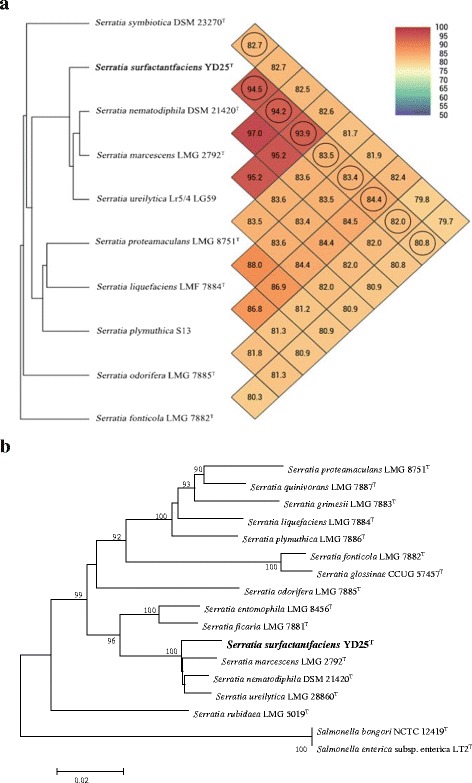
Taxonomic classification by genome-wide comparative analysis of YD25^T^. **a** Heatmap generated with OrthoANI values calculated from the OAT software. Pairwise OrthoANI calculations among the selected *Serratia* genomes. A comparison with genome sequences of the strains of other established *Serratia* species showed that strain YD25^T^ shared < 95 % similarity: 94.5 % with *S. nematodiphila*, 94.2 % with *S. marcescens*, 93.9 % with *S. ureilytica*, 84.4 % with *S. plymuthica*, 83.5 % with *S. proteamaculans*, 83.4 % with *S. liquefaciens*, 82.7 % with *S. symbiotica*, 82.0 % with *S. odorifera* and 80.8 % with *S. fonticola*. **b** Neighbor-joining tree showing the phylogenetic relationships of strains YD25^T^ and phylogenetically related reference strains based on concatenated partial *rpo*B, *atp*D, *gyr*B and *inf*B gene sequences. Bootstrap analysis (1000 resamplings) was used to evaluate the topology of the NJ tree, and the bootstrap values >70 % are displayed at branch points. Bar, 0.02 substitutions per nucleotide position

The ten genomes were analyzed by pairwise genome calculations using OrthoANI, and the results are shown in Fig. 7a. In all cases, these OrthoANI values clearly indicate that YD25^T^ has OrthoANI less than 95 % of other closely related *Serratia* species, representing a different genomo-species from that currently classified within the group and again confirming species-level distinctiveness. Although the genome sequence of the *S. ureilytica* and *S. plymuthica* type strains is unavailable, the two species may belong to the group represented by Lr5/4 LG59 and S13. Although the genome sequences of the remaining species *Serratia* is not publicly available, the comparison results are unaffected because these species give the low 16S rRNA identity compared with YD25^T^. DDH values between strain YD25^T^ and *S. nematodiphila* DSM 21420^T^, *S. marcescens* LMG 2792^T^, *S. ureilytica* Lr5/4 LG59, *S. proteamaculans* LMG 8751^T^, *S. liquefaciens* LMG 7884^T^, *S. plymuthica* S13, *S. symbiotica* DSM 23270^T^, *S. odorifera* LMG 7885^T^, *S. fonticola* LMG 7882^T^ were < 58 % (Table 3), confirming that strain YD25^T^ represents a novel species of the genus *Serratia*.

**Table 3:** DNA-DNA hybridization values between strain YD25^T^ and strains of phylogenetically related species. The parameter is calculated using the genome-to-genome distance calculator (GGDC) available online with formula 2 method

Strains	Identities/HSP length	Probability > 70 %	Probability > 79 %
*S. nematodiphila* DSM 21420^T^	57.90 % ± 2.77	45.06 %	35.51 %
*S. marcescens* LMG 2792^T^	56.40 % ± 2.75	39.98 %	8.95 %
*S. ureilytica* Lr5-4 LG59	55.30 % ± 2.72	35.95 %	7.86 %
*S. plymuthica* S13	28.30 % ± 2.43	0.05 %	0.02 %
*S. proteamaculans* LMG 8751^T^	27.20 % ± 2.43	0.03 %	0.02 %
*S. symbiotica* DSM 23270^T^	27.00 % ± 2.42	0.03 %	0.01 %
*S. liquefaciens* LMG 7884^T^	27.00 % ± 2.42	0.03 %	0.01 %
*S. odorifera* LMG 7885^T^	25.10 % ± 2.40	0.01 %	0.01 %
*S. fonticola* LMG 7882^T^	24.60 % ± 2.40	0.01 %	0 %

In addition, once the gene sequences were retrieved from the genomes, an MLSA approach was performed using the concatenated sequences of the four housekeeping genes (*rpo*B, *gyr*B, *inf*B and *atp*D; total alignment length 2636 bp). Strain YD25^T^ showed 98 % concatenated partial *rpo*B, *gyr*B, *inf*B and *atp*D gene sequence similarity to the types of the most phylogenetically related species, *S. nematodiphila* and *S. marcescens* (Additional file 10: Table S5). For the analysis, strain YD25^T^ formed an independent branch and was clearly distant from the core species of the group (*S. nematodiphila*, *S. marcescens* and *S. ureilytica*) (Fig. 7b). The bootstrap values indicated a very stable branching order and mirrored the results observed with the phylogenetic reconstruction analyses based on 16S rRNA. Individual *rpo*B gene sequences were also analyzed, and the tree agreed in the topology and showed a consistent branching order with the tree of the remaining four housekeeping genes (Additional file 11: Figure S6). These results reinforced the observation that YD25^T^ represented a novel species of the group.

### Biochemical and physiological features of YD25^T^

The biochemical, physiological, and morphological characteristics of strain YD25^T^ are summarized in the species description (Additional file 12), and a comparison of these features with the closely related species is presented in Table 4. In contrast to *S. nematodiphila* DSM 21420^T^ or *S. marcescens* LMG 2792^T^, strain YD25^T^ can use adonitol, D-arabinose, D-xylose and melibiose but cannot utilize D-lactose or raffinose and is methyl red test-positive. Importantly, three highly specific features of the YD25^T^ taxon are the presence of urease activity, absence of lipase activity and failure of lactate assimilation. The presence of urease activity may be explained by the presence of gene coding for α, β, and γ subunits of urease, and the absence of lipase activity may be explained by the absence of gene coding for lipase, as observed in the genome of YD25^T^. Concerning the absence of lactate assimilation, we observed that the genome of YD25^T^ does not contain the lactate oxidase encoding gene. These biochemical and physiological features distinguish YD25^T^ from its close relatives.

**Table 4:** Biochemical and physiological characteristics of strain YD 25^T^ and phylogenetically related reference type strains in the genus *Serratia*

Characteristic	1	2	3	4	5	6	7
Fluorescence	+	-	-	-	-	NA	-
Pigment production	+	+	-	-	+	-	+
Methyl red test	-	-	+	+	-	+	+
Urease	-	-	+	-	-	-	+
Lipase (Tween 80)	+	+	+	-	+	+	-
Adonitol	+	-	+	-	+	+	+
D-Lactose	+	-	-	+	+	+	+
D-Arabinose	+	-	-	+	+	-	+
Raffinose	+	-	-	+	+	+	-
D-Xylose	+	-	+	+	+	+	+
Melibiose	+	-	+	+	+	+	+
Lactate	+	+	+	+	+	+	-
D-Serine	+	+	+	-	-	NA	+
L-Ornithine	+	+	+	+	-	+	+
Alanine	+	+	+	+	-	+	+
L-Proline	+	+	+	+	+	+	+
Phenylalanine	-	+	+	-	-	-	-
DNA G + C content (mol%)	59.52	57.5-60	60	NA	NA	59.6	59.62

1, *S. nematodiphila* DSM 21420^T^; 2, *S. marcescens* LMG 2792^T^; 3, *S. ureilytica* LMG 22860^T^; 4, *S. odorifera* LMG 7885^T^; 5, *S. rubidaea* LMG 5019 ^T^; 6, *S. ficaria* LMG 7881^T^; 7, strain YD25^T^. +, positive; -, negative; NA, data not available

The comparative fatty acid compositions of YD25^T^ and the type strains of phylogenetically related reference strains are given in Table 5. The major fatty acid in strain YD25^T^ is C_16:0_, which was in agreement with the profiles of most *Serratia* species. More importantly, YD25^T^ contained the major fatty acid C_16:0_ (30.73 %), which was the same as those of *S. nematodiphila* DSM 21420^T^, *S. marcescens* LMG 2792^T^, and *S. ureilytica* LMG 22860^T^. However, the relative proportions of the major fatty acids of YD25^T^ were significantly different from the three close relative strains. In particular, the predominant major fatty acid of YD25^T^ was C_16:0_, C_18:1_ *ω7c* and C_17:0_ cyclo, while those of the other three strains were C_16:0_, C_17:0_ cyclo and C_19:0_ cyclo *ω8c*; C_16:0_, C_18:1_ *ω7c* and C_17:0_ cyclo; and C_16:0_, C_18:1_ *ω7c* and summed feature 1 (comprises C_14:0_ 3-OH/C_16:1_ iso I), respectively. In addition, there were significant differences in the percentages of other fatty acids between YD25^T^ and each of the latter three phylogenetically related reference type strains. The most marked differences between YD25^T^ and the abovementioned three reference type strains tested concerned the fatty acids summed feature 2 (comprises C_16:1_ *ω7c*/C_16:1_ *ω6c*) and C_19:0_ cyclo *ω8c*, which were present in YD25^T^ and absent from large amounts in most tested strains. However, the YD25^T^ presented significantly higher amounts of C_14:0_ 2-OH and C_12:0_ 2-OH and significantly lower amounts of C_14:0_ compared to the latter strains.

**Table 5:** Cellular fatty acid compositions (%) of strain YD25^T^ and phylogenetically related reference type strains in the genus *Serratia*

Fatty acid	1	2	3	4	5	6	7
C_10:0_ 3-OH	tr	ND	ND	ND	ND	ND	tr
C_12:0_	2.47	1.46	1.4	2.95	4.28	5.80	1.62
C_12:0_ 2-OH	tr	tr	tr	tr	ND	1.51	1.73
C_12:0_ 3-OH	tr	ND	ND	ND	ND	ND	tr
C_14:0_	8.4	6.94	6.28	7.4	6.52	8.14	3.39
C_14:0_ 2-OH	1.02	2.05	2.21	ND	ND	ND	2.89
C_15:0_	tr	ND	tr	ND	1.31	ND	ND
C_16:0_	34.76	31.86	31.01	30.74	17.98	24.51	30.73
C_16:0_ *ω5c*	ND	ND	ND	ND	ND	ND	tr
C_17:0_ cyclo	20.03	11.68	2.69	4.96	ND	12.45	14.56
C_17:0_	ND	tr	tr	ND	tr	2.26	tr
C_18:1_ *ω7c*	1.67	16.88	17.89	14.4	11.39	9.56	15.29
C_19:0_ iso	tr	ND	ND	ND	ND	ND	tr
C_19:0_ cyclo *ω7c*	17.24	1.24	ND	tr	ND	ND	ND
C_19:0_ cyclo *ω8c*	ND	ND	tr	ND	ND	1.68	2.42
Summed features^a^							
1	7.56	8.44	8.01	8.98	4.11	14.54	8.36
2	ND	ND	ND	ND	ND	ND	15.78

^a^Summed feature 1 comprises C_14:0_ 3-OH/C_16:1_ iso I; summed feature 2 comprises C_16:1_ *ω7c*/C_16:1_ *ω6c*

Strains: 1, *S. nematodiphila* DSM 21420^T^; 2, *S. marcescens* LMG 2792^T^; 3, *S. ureilytica* LMG 22860^T^; 4, *S. odorifera* LMG 7885^T^; 5, *S. rubidaea* LMG 5019 ^T^; 6, *S. ficaria* LMG 7881^T^; 7, strain YD25^T^. tr, Trace amount (<1.0 %); ND, not detected

In combination with the unique features of its genomic sequence, the biochemical, physiological, and morphological characteristics of strain YD25^T^ confirmed that it represents a distinct and separate species within the genus *Serratia*.

## Discussion

*Serratia* belongs to *Enterobacteriaceae* and can produce various extracellular products, including lipases, chitinases, nucleases, proteases, and various antimicrobial secondary metabolites [65]. This has been linked to the ability of *Serratia* strains to colonize a wide range of ecological niches [4]. Prodigiosin is the most characterized antimicrobial compound in *Serratia* strains [66]. Serrawettin W2 acts as a biosurfactant to reduce the surface tension of the environment to allow bacterial spreading across surfaces and has been shown to possess antimicrobial activity [67]. Furthermore, *Serratia* species produce a wealth of volatile organic compounds, such as sodorifen, dimethyl trisulfide, methanethiol, and terpenoids, which might influence the growth of phytopathogenic fungi or bacteria [68, 69]. From these aspects, it was established that YD25^T^ is an unusual antagonistic bacterium through secretion of a variety of different antimicrobial substances.

YD25^T^ has a special trait of simultaneous production of both serrawettin W2 and prodigiosin. Such a co-production feature has not been reported in other *Serratia* sp. strains. For serrawettins, serrawettin W1 was produced by pigmented *S. marcescens* ATCC 274 [70], *S. marcescens* CH-1 [71], *S. marcescens* NS-38 [72], *S. marcescens* WW4 [73] and so on, while serrawettin W2 and W3 were produced by nonpigmented *Serratia* strains [11, 73]. Moreover, *Serratia* strains that could produce more than one type of serrawettin have not been recognized. Serrawettin W2 was originally isolated from nonpigmented *S. marcescens* NS 25 [16]. *S. marcescens* Db10 is an insect pathogen that could produce serrawettin W2 but lacks the genes to produce prodigiosin [6]. There are pigment genes, and a 2631 bp sequence of *swr*A was found in *S. marcescens* W2.3 isolated from diseased tilapia fish; nevertheless, the related products have not been isolated and identified [74]. To date, YD25^T^ is the first reported *Serratia* strain that could concurrently produce the antibiotic secondary metabolite, prodigiosin and serrawettin W2 and also produce a spectrum of putative serrawettin W2 analogues simultaneously.

Cyclic lipopeptides exhibiting antibacterial activities, such as massetolide, viscosin, syringomycin, arthrofactin, and orfamide, have emerged as promising candidates for the development of new antibiotics [75, 76]. Serrawettin W2 is a broad spectrum lipopeptide that could suppress both Gram-positive and Gram-negative bacteria in our research. Furthermore, serrawettin W2 has cytotoxicity on cancer cells and shows selectivity for different cancer cell lines. Therefore, serrawettin W2 has attracted interest as a potentially valuable antibiotic. At present, the available biosynthetic gene cluster for serrawettin W2 has not been identified, unless the 2.8 kb incomplete *swr*A is involved in the biosynthesis of the serrawettin W2 NRPS gene in *S. marcescens* MG1 [73] and *S. marcescens* A88copa13 [74]. In our study, a gene cluster of serrawettin W2 biosynthesis in YD25^T^ was directed by a hybrid PKS-NRPS system, and domain functions and a biosynthesis pathway were proposed. Although the formation of the C_10_ unit fatty acid chain by the PKS remains unclear; *Serratia*, a genetically tractable bacterium, has allowed a detailed dissection of how the secondary metabolite is biosynthesized. Furthermore, modules and domains of PKS and NRPS enzymes within the serrawettin W2 biosynthetic gene cluster offers potential future exploitation for the generation and synthesis of useful and modified natural products.

In a complete AI-1 system, the *lux*I/R homologs interact with each other [28]. The LuxI-type proteins synthesize various AHL signals, and LuxR-type transcriptional regulatory proteins bind their cognate signal and in turn induce/repress multiple gene expression accordingly [27]. However, *lux*I and *lux*R genes do not occur in pairs in the YD25^T^ genome. The unpaired *lux*R gene has also been reported in the *S. marcescens* W2.3 genome [74]. *Car*R as a solo *lux*R, regulates the production of two secondary metabolite antibiotics, prodigiosin and carbapenem, in *S. marcescens* ATCC 39006 [77]. However, the sequence similarities at the amino acid level of *sfc*R and *sfs*R with *car*R are only 37 % and 40 %, respectively; therefore, the two solo *lux*R may be classified as different transcriptional protein families from *car*R. Moreover, a complete AI-2-mediated system is found in the YD25^T^ genome. Interestingly, comparative sequence analysis of the *lsr* clusters in 43 other *Serratia* isolates, including the *lux*S and complete *lsr* gene cluster in the genome sequence, also revealed the consistent arrangement order of ORF with the one in YD25^T^. Specifically, the *lsr* genes are all in one polycistron in other *Enterobacteriaceae* bacteria, such as *S. typhimurium* [78], *E. coli* [79], and *E. fergusonii* [33], while *lsr*BACDEF, *lsr*GK, and *lsr*R are dispersed in the *Serratia* genome sequence. In addition, due to the differences among genera, the sequence similarity of LsrBACDEF and LsrR of *Serratia* show a lower degree with their homologues in other *Enterobacteriaceae* bacteria. Based on phylogenetic analysis with concatenated amino acid sequences of 10 proteins (LsrABCDEFGKR and LuxS), we observed a significant correlation between the *lsr* operon and secondary metabolites, prodigiosin and serrawettin W2, of *Serratia* isolates in general. Therefore, we conclude that AI-2 regulates the biosynthesis of the two compounds. AI-2-mediated systems have been shown to regulate various secondary metabolites produced by strains of *Serratia* [80], and it will be interesting to study whether AI-2 is directly involved in regulating the expression of the *pig* and *swr* gene clusters in *Serratia*.

The 16S rRNA gene sequence of YD25^T^ was related most closely to *S. nematodiphila* DSM 21420^T^ [63] and *S. marcescens* LMG 2792^T^ [2, 81]. The phylogenetic reconstruction based on 16S rRNA was of very low resolution due to the high interspecies similarity of the 16S rRNA gene sequences. However, the biochemical, physiological and morphological features of these three strains distinguishes YD25 ^T^ from the other two strains. In addition, the results of OrthoANI, GGDC, and MLSA analysis based on the analysis of genome sequences strongly support that YD25^T^ is different from other species of *Serratia*. Moreover, phylogenetic analysis based on concatenated Lsr and LuxS proteins revealed that YD25^T^ forms an independent branch and is clearly distant from the strains that solely produce either prodigiosin or serrawettin W2. Combining both classical taxonomic analysis and genomic sequence analysis, we propose that YD25^T^ is a novel species of the genus *Serratia*, to which the name *Serratia surfactantensis* sp. nov. is proposed.

## Conclusions

The phylogenetic, genotypic and phenotypic data support that strain *Serratia* sp. YD25^T^ (=CCTCC AB 2015384; =KCTC 42987) is a member of a novel and previously uncharacterized *Serratia* species; the name *Serratia surfactantensis* sp. nov. is proposed. The genomic sequence and metabolite analysis of *S. surfactantfaciens* YD25^T^ indicates that this strain can be further explored for the production of useful metabolites. *S. surfactantfaciens* YD25^T^ can simultaneously produce both prodigiosin and serrawettin W2, therefore unveiling its genomic sequence benefits using this novel species as a model system for studying the biosynthesis regulation of these two useful compounds by the QS system.

## Additional files

Additional file 1: Figure S1. The morphological characteristics of YD25^T^. (A) A circular irregular margin morphology of YD25^T^ after incubation for 48 h at 30 °C on KB agar; (B) Scanning electron micrograph of YD25^T^, Bar, 100 nm; (C) Swimming colony of YD25^T^ after incubation for 48 h at 30 °C on 0.3 % agar KB swimming plate; (D) Swarming colony of YD25^T^ after incubation for 48 h at 30 °C on 0.5 % agar LB swarming plate. (DOCX 434 kb)
Additional file 2: Figure S2. Antagonistic activities of YD25^T^ against fungi. (A) Visualization of the YD25^T^-*E. turcicum*, YD25^T^-*F. oxysporum*, YD25^T^-*A. alternata* and YD25^T^-*C. sativus* confrontation assays, 3-7 days after inoculation of fungi in the presence of YD25^T^ or in its absence. (B) The biofilm culture assay of *M. luteus* co-cultured with and without YD25^T^ at 30 °C; (C) The biofilm culture assay of *R. solanacearum* co-cultured with and without YD25^T^ at 30 °C. Numbers show an average of three replications, and error bars show standard errors of the means. (DOCX 826 kb)
Additional file 3: Table S1.
^1^H and ^13^C NMR chemical shift assignments of sw-5 (serrawettin W2). (DOCX 16 kb)
Additional file 4: Figure S3. Circular map of the YD25^T^ chromosome. From outside to the center: genes on forward strand, genes on reverse strand, GC content, GC skew. (DOCX 297 kb)
Additional file 5: Table S2. Deduced functions and homologues of the gene products from the *pig* clusters. The relatedness of each YD25^T^ pig protein to its homologue in *S. marcescens* ATCC 274, *S. plymuthica* AS 13, and *Serratia* sp. ATCC 39006 is shown in the comparison of *Serratia* pig protein percentage sequence identity column. (DOCX 15 kb)
Additional file 6: Table S3. Deduced functions and homologues of the gene products from the *lsr* clusters. The relatedness of each YD25^T^ Lsr protein to its homologue in *Salmonella typhimurium* LT2, *E. coli* K-12, and *E. fergusonii* ATCC 35469 is shown in the comparison of Lsr protein percentage sequence identity column. (DOCX 15 kb)
Additional file 7: Figure S4. Phylogenetic analyses of concatenated gene sequences in the *lsr* operon and *luxS*. Bootstrap analysis (1000 resamplings) was used to evaluate the topology of the NJ tree. Bar, 0.01 substitutions per nucleotide position. All sequences were retrieved from the NCBI database or in published genome projects. Boxes represent the strains in which the biosynthetic gene cluster of prodigiosin (red) or serrawettin W2 (blue) are observed in the genome. (DOCX 241 kb)
Additional file 8: Table S4. The less than 98.7 % 16S rRNA gene sequence similarities between YD25^T^ and type strains of phylogenetically related species. (DOCX 12 kb)
Additional file 9: Figure S5. Neighbor-joining tree showing the phylogenetic relationships of YD25^T^ and phylogenetically related reference strains based on 16S rRNA gene sequences. Bootstrap analysis (1000 resamplings) was used to evaluate the topology of the NJ tree, and the bootstrap values > 70 % are displayed at branch points. Bar, 0.005 substitutions per nucleotide position. (DOCX 155 kb)
Additional file 10: Table S5. 16S rRNA, *rpo*B, *atp*D, *gyr*B and *inf*B gene sequence similarities between YD25^T^ and type strains of phylogenetically related species. (DOCX 13 kb)
Additional file 11: Figure S6. Neighbor-joining tree showing the phylogenetic relationships of YD25^T^ and phylogenetically related reference strains based on *rpo*B gene sequences. Bootstrap analysis (1000 resamplings) was used to evaluate the topology of the NJ tree, and the bootstrap values > 50 % are displayed at branch points. Bar, 0.01 substitutions per nucleotide position. (DOCX 148 kb)
Additional file 12: Description of *Serratia surfactantfaciens* sp. nov. (DOCX 16 kb)

## Abbreviations

A: Adenylation domain
AHL: N-acyl-L-homoserine lactone
AI-1: Autoinducer-1
AI-2: Autoinducer-2
ANI: Average nucleotide identity
AT: Acyltransferase
C: Condensation domain
CFS: Cell-free supernatant sample
DDH: DNA-DNA hybridization
ESI: Electrospray ionization
FA: Fatty acid
GGDC: Genome to genome distance calculator
KR: Keto reductase
KS: Ketosynthase
MLSA: Multi-locus sequence analysis
MLST: Multi-locus sequence typing
NCBI: National Center for Biotechnology Information
NRPS: Non-ribosomal peptide synthetases
ORF: Open reading frame
PKS: Hybrid polyketide synthases
QS: Quorum sensing
T: Thiolation domain
TE: Thioesterase domain
