# Supplementary figures and images for: Analysis of the genomic sequences and metabolites of Serratia surfactantfaciens sp. nov. YD25T that simultaneously produces prodigiosin and serrawettin W2
Source: BMC Genomics. 2016 Nov 3;17:865. doi: 10.1186/s12864-016-3171-7 (PMC5094094; doi:10.1186/s12864-016-3171-7)

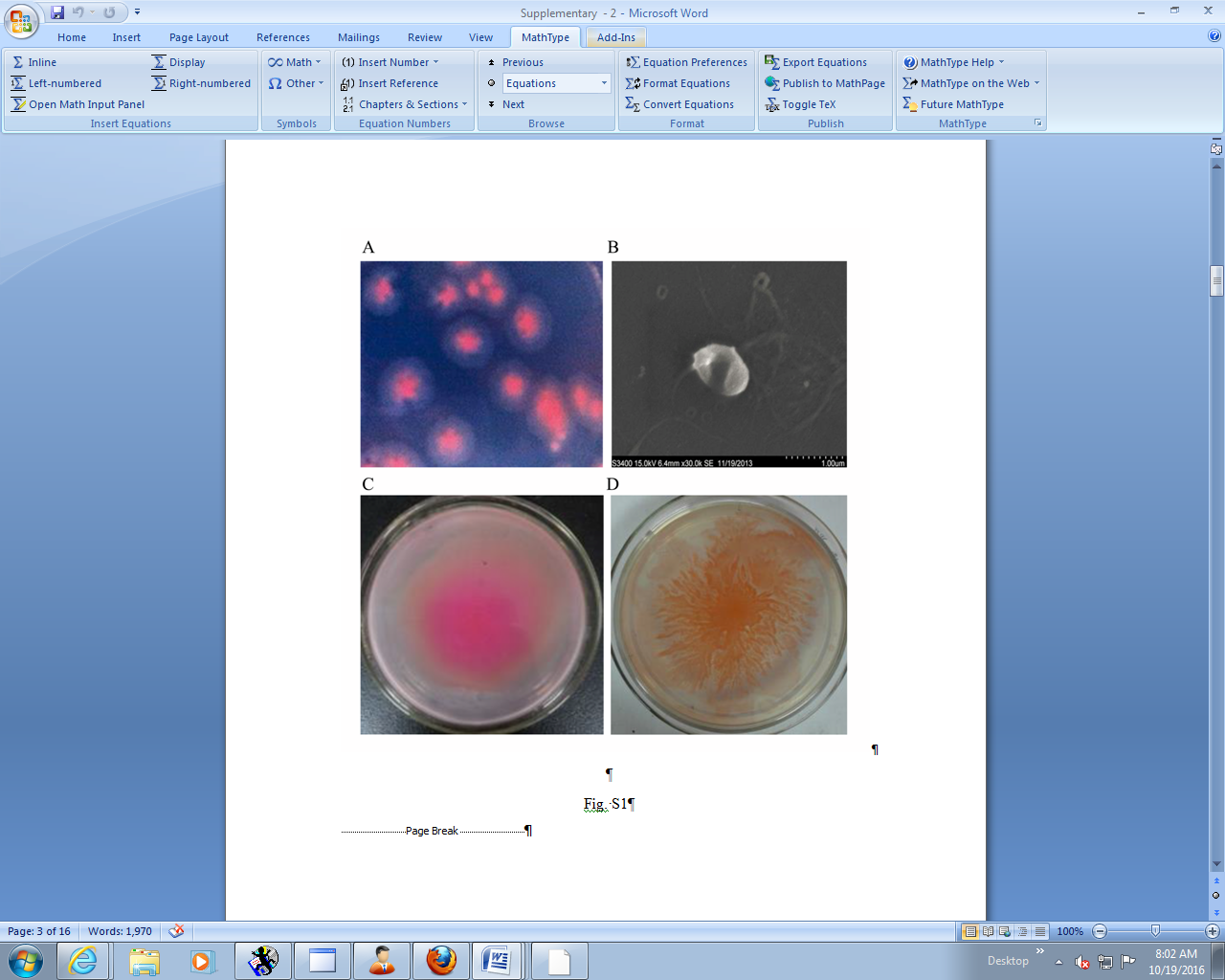

Supplement: Additional file 1: Figure S1. — The morphological characteristics of YD25T. (A) A circular irregular margin morphology of YD25T after incubation for 48 h at 30 °C on KB agar; (B) Scanning electron micrograph of YD25T, Bar, 100 nm; (C) Swimming colony of YD25T after incubation for 48 h at 30 °C on 0.3 % agar KB swimming plate; (D) Swarming colony of YD25T after incubation for 48 h at 30 °C on 0.5 % agar LB swarming plate. (DOCX 434 kb) [file 12864_2016_3171_MOESM1_ESM.docx]

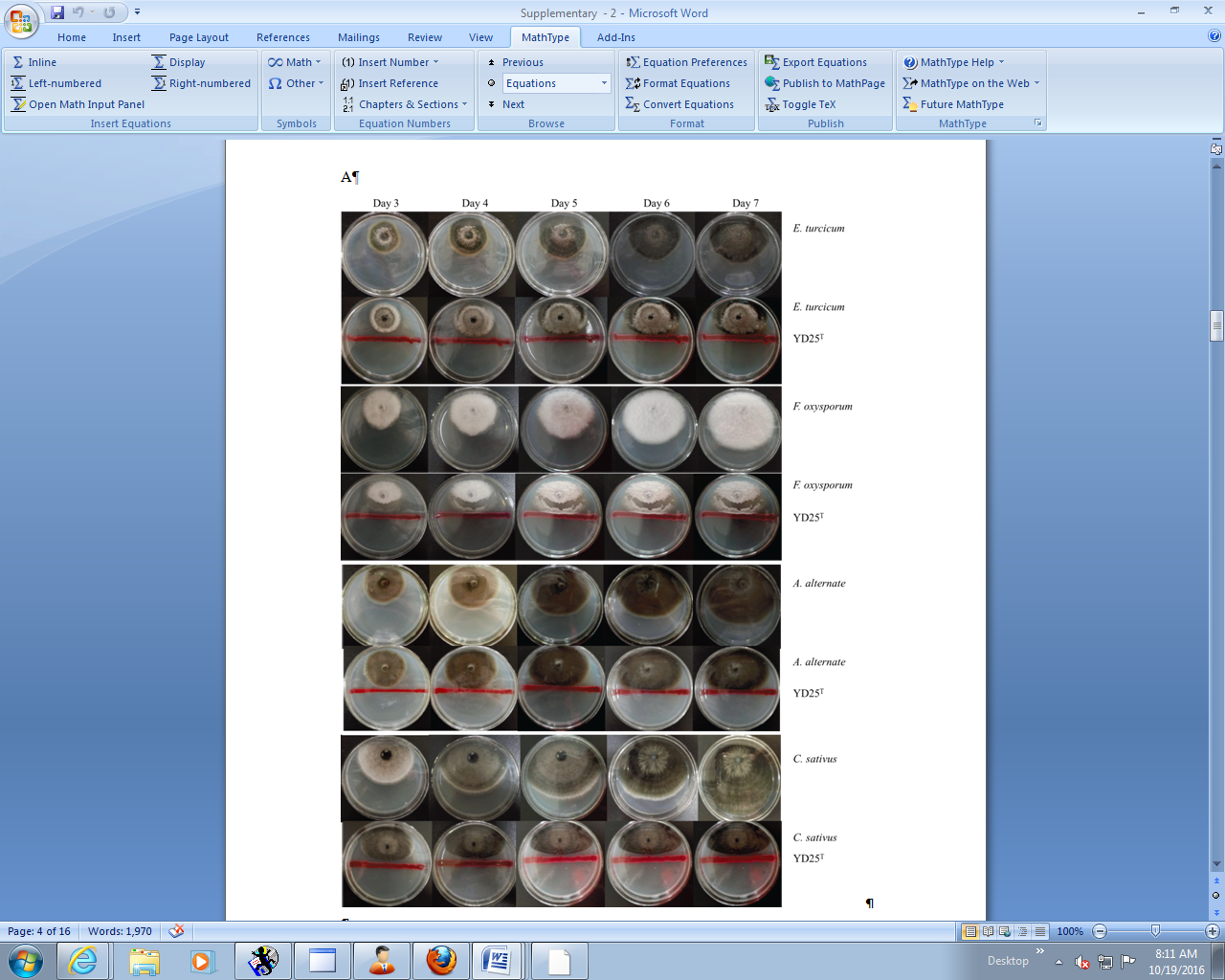


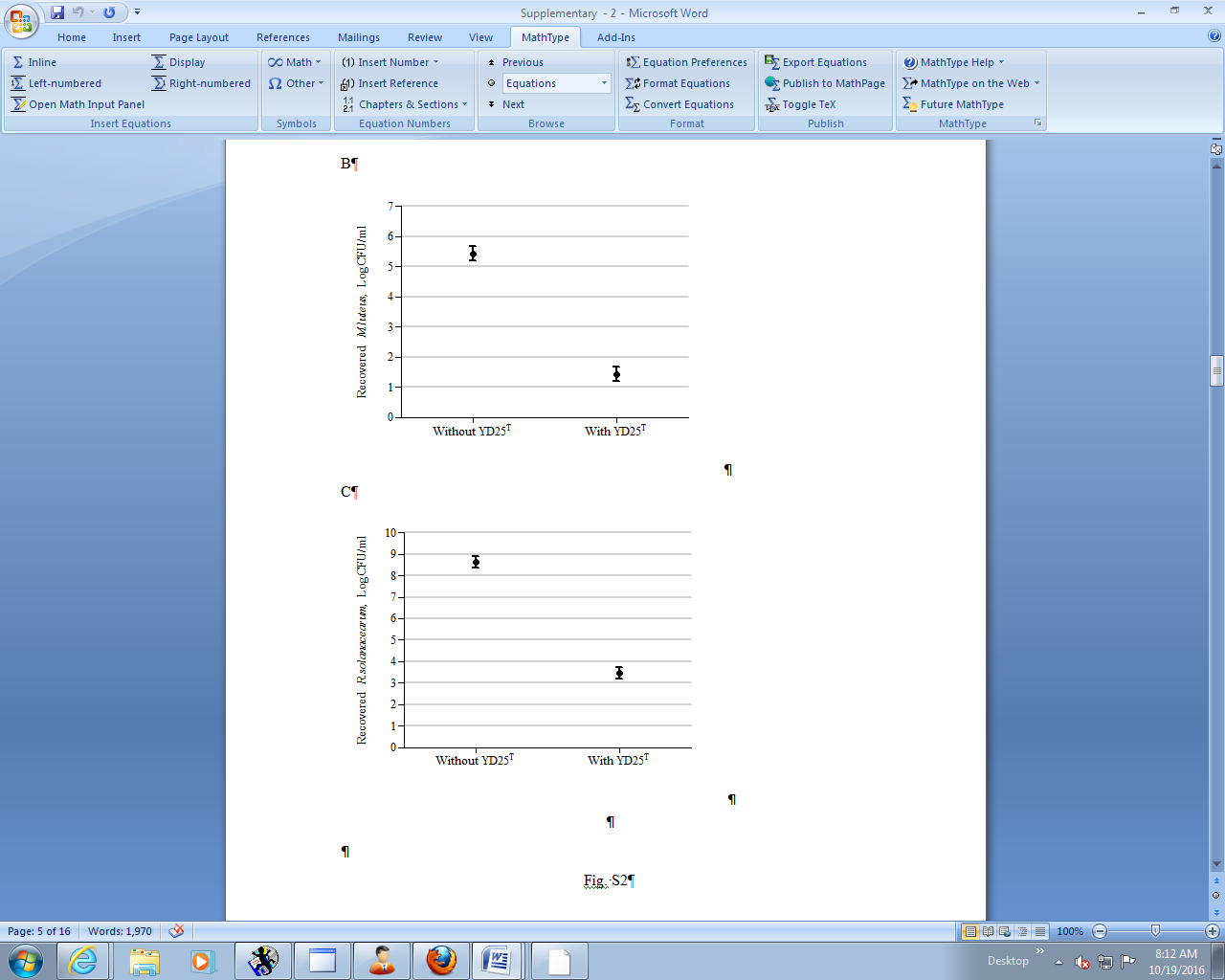

Supplement: Additional file 2: Figure S2. — Antagonistic activities of YD25T against fungi. (A) Visualization of the YD25T-E. turcicum, YD25T-F. oxysporum, YD25T-A. alternata and YD25T-C. sativus confrontation assays, 3-7 days after inoculation of fungi in the presence of YD25T or in its absence. (B) The biofilm culture assay of M. luteus co-cultured with and without YD25T at 30 °C; (C) The biofilm culture assay of R. solanacearum co-cultured with and without YD25T at 30 °C. Numbers show an average of three replications, and error bars show standard errors of the means. (DOCX 826 kb) [file 12864_2016_3171_MOESM2_ESM.docx]

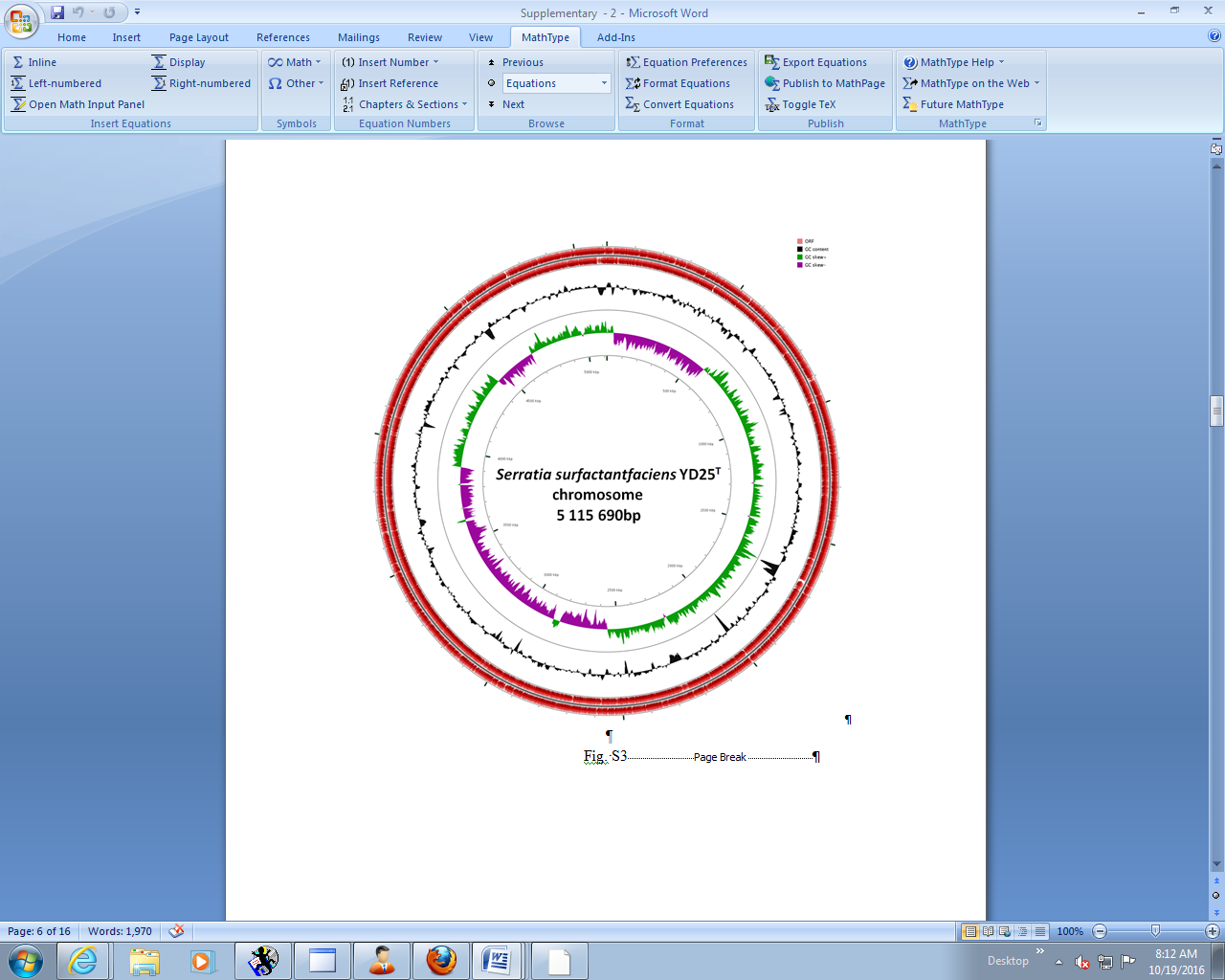

Supplement: Additional file 4: Figure S3. — Circular map of the YD25T chromosome. From outside to the center: genes on forward strand, genes on reverse strand, GC content, GC skew. (DOCX 297 kb) [file 12864_2016_3171_MOESM4_ESM.docx]

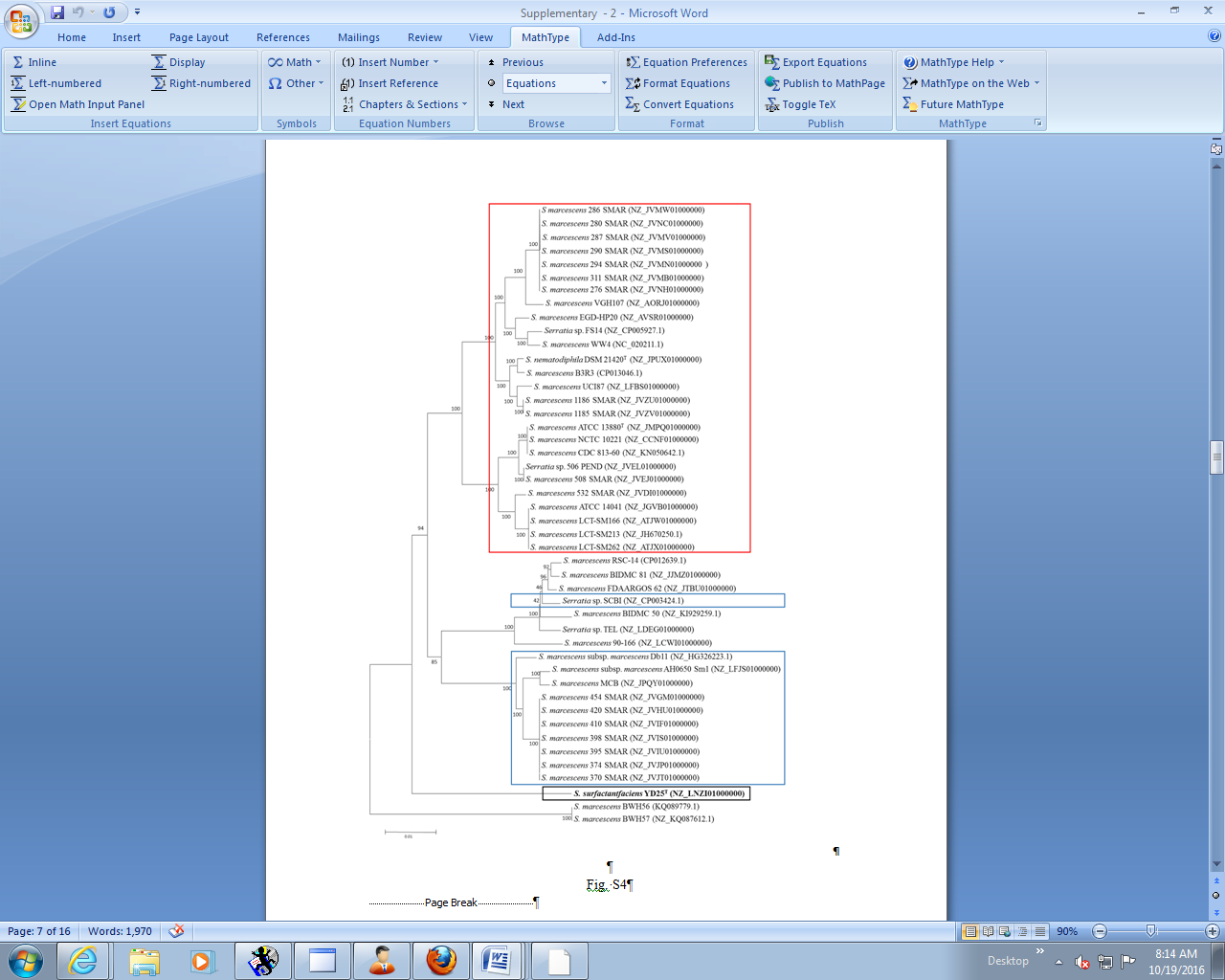

Supplement: Additional file 7: Figure S4. — Phylogenetic analyses of concatenated gene sequences in the lsr operon and luxS. Bootstrap analysis (1000 resamplings) was used to evaluate the topology of the NJ tree. Bar, 0.01 substitutions per nucleotide position. All sequences were retrieved from the NCBI database or in published genome projects. Boxes represent the strains in which the biosynthetic gene cluster of prodigiosin (red) or serrawettin W2 (blue) are observed in the genome. (DOCX 241 kb) [file 12864_2016_3171_MOESM7_ESM.docx]

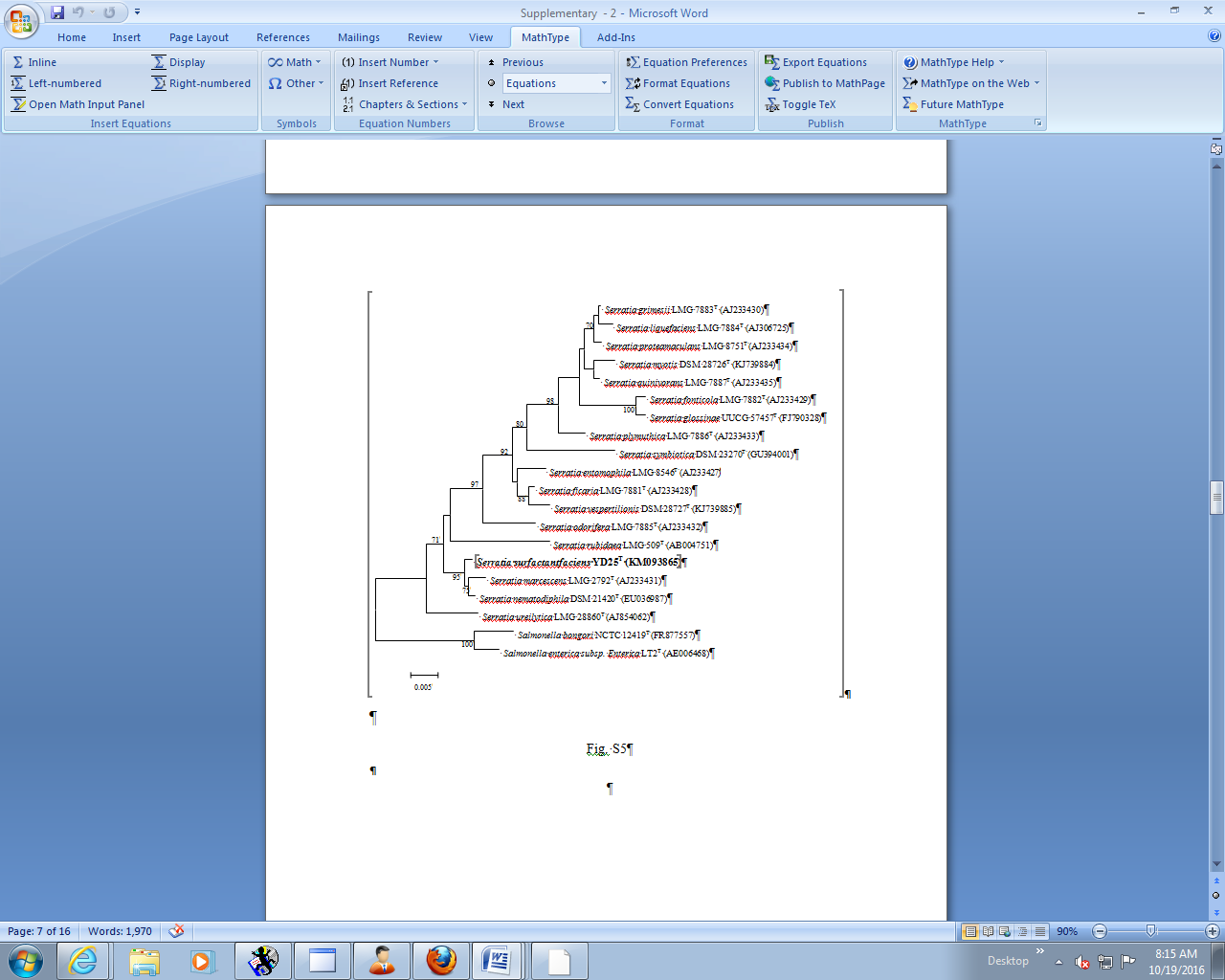

Supplement: Additional file 9: Figure S5. — Neighbor-joining tree showing the phylogenetic relationships of YD25T and phylogenetically related reference strains based on 16S rRNA gene sequences. Bootstrap analysis (1000 resamplings) was used to evaluate the topology of the NJ tree, and the bootstrap values > 70 % are displayed at branch points. Bar, 0.005 substitutions per nucleotide position. (DOCX 155 kb) [file 12864_2016_3171_MOESM9_ESM.docx]

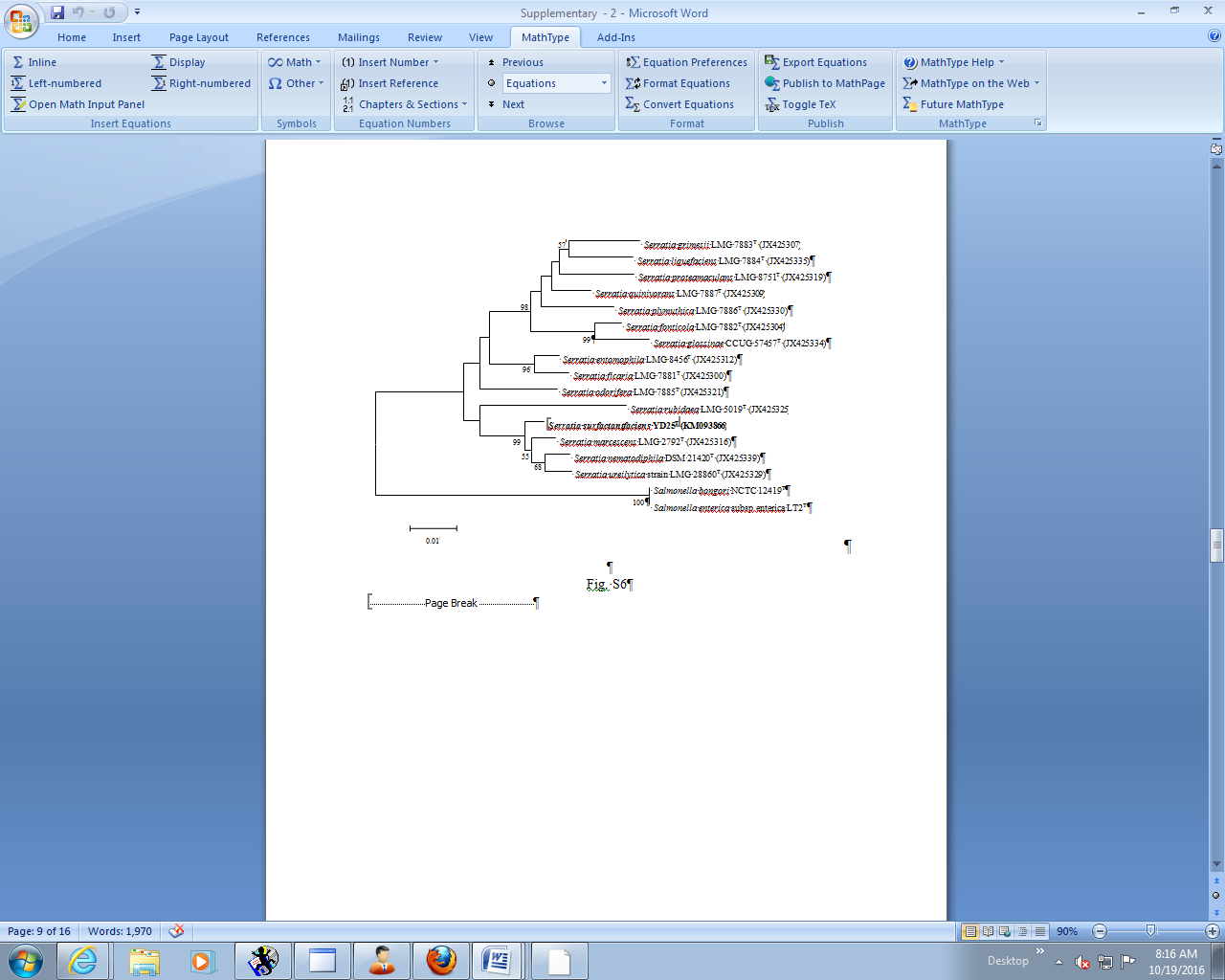

Supplement: Additional file 11: Figure S6. — Neighbor-joining tree showing the phylogenetic relationships of YD25T and phylogenetically related reference strains based on rpoB gene sequences. Bootstrap analysis (1000 resamplings) was used to evaluate the topology of the NJ tree, and the bootstrap values > 50 % are displayed at branch points. Bar, 0.01 substitutions per nucleotide position. (DOCX 148 kb) [file 12864_2016_3171_MOESM11_ESM.docx]
