# Supplementary material for: Analysis of the genomic sequences and metabolites of Serratia surfactantfaciens sp. nov. YD25T that simultaneously produces prodigiosin and serrawettin W2
Source: BMC Genomics. 2016 Nov 3;17:865. doi: 10.1186/s12864-016-3171-7 (PMC5094094; doi:10.1186/s12864-016-3171-7)
Supplement: Additional file 3: Table S1. — 1H and 13C NMR chemical shift assignments of sw-5 (serrawettin W2). (DOCX 16 kb) [file 12864_2016_3171_MOESM3_ESM.docx]

**Table S1. ^1^H and ^13^C NMR chemical shift assignments of sw-5 (serrawettin W2).**

| Residue | δc | δ_H_(ppm) | Literature^a^ | |
| --- | --- | --- | --- | --- |
|  |  |  | δc | δ_H_(ppm) |
| **Leu（1）** |  |  |  |  |
| NH | - | 7.83 (d, 8.6) |  |  |
| C_α_ | 50.64 | 4.36(m) |  |  |
| C_β_ | 40.32 | 1.46 – 1.41(m), | 24.8^b^ |  |
|  |  | 1.19 – 1.11 (m) |  |  |
| C_γ_ | 23.71 | 1.65(m) | 23.4 | 1.64m |
| C_δ_ | 20.94 | 0.90–0.76(m) |  |  |
|  | 23.31 | 0.90–0.76(m) |  |  |
| α-CO | 172.09 | - |  |  |
| **Ser（2）** |  |  |  |  |
| NH | - | 8.09 (d, 8.4) |  |  |
| C_α_ | 56.61 |  |  |  |
| C_β_ | 61.11 | 3.63 (dd,10.9, 6.5), | 61.3 | 3.63(11.0,6.4) |
|  |  | 3.56 (dd, 10.8, 6.7) |  | 3.56(11.0,6.8) |
| α-CO | 170.58 | - |  |  |
| β-OH | - | 5.05 (s) |  |  |
| **Thr（3）** |  |  |  |  |
| NH | - | 8.04 (d, 8.4) |  |  |
| C_α_ | 58.39 | 4.11(dd,8.5, 3.0) | 58.5 | 4.11(8.4,2.9) |
| C_β_ | 65.35 | 4.25 (d, 7.6) | 65.4 | 4.24 m |
| C_γ_ | 20.12 | 0.98 (d, 6.3) | 20.2 | 0.97(6.4) |
| α-CO | 169.47 | - |  |  |
| β-OH | - | 5.13 (s) |  |  |
| **Phe（4）** |  |  |  |  |
| NH |  | 7.46 (d,6.9) |  | 7.46(7.0) |
| C_α_ | 53.50 | 4.50 – 4.41(m) |  |  |
| C_β_ | 37.09 | 3.14(dd,13.8,4.5) | 37.2 | 3.14(13.7,4.5) |
|  |  | 2.90(dd,13.8,7.2) |  | 2.89(13.7,7.2) |
| γ | 136.74 |  |  |  |
| δ | 128.39 | 7.14 (m) |  |  |
| ε | 129.05 | 7.09–7.05(m) |  |  |
| ζ | 126.39 | 7.14(m) |  |  |
| α-CO | 170.53 |  |  |  |
| **Ile（5）** |  |  |  |  |
| NH | - | 8.51 (d, 6.6) |  | 8.49(6.4) |
| C_α_ | 57.51 | 3.79 – 3.75 (m) | 57.6 | 3.76(8.2,6.8) |
| C_β_ | 34.57 | 1.78 (qd,7.7, 4.1) | 34.7 | 1.77m |
| C_γ_ | 24.73 | 1.33-1.46(m) | 40.4^b^ |  |
|  |  | 1.11-1.19(m) |  |  |
| C_δ_ | 10.08 | 0.90–0.76(m) |  |  |
| β-CH_3_ | 15.25 | 0.90–0.76(m) |  |  |
| **OHDa** |  |  |  |  |
| C1 | 168.8 | - |  |  |
| C2 | 40.06 | 2.64(dd,14.4,3.2 ), | 39.0 | 2.64(14.5,3.0) |
|  |  | 2.37 (dd,14.4,5.9 ) |  | 2.36(14.5,5.9) |
| C3 | 71.67 | 4.93(m） | 71.8 | 4.92m |
| C4 | 31.87 | 1.53(m) | 31.1 | 1.53br m |
| C5 | 24.73 | 1.24 m) |  | 1.23br |
| C6 | 28.54 | 1.24 (m) |  | 1.23br |
| C7 | 28.33 | 1.24 (m) |  | 1.23br |
| C8 | 31.02 | 1.24 (m) |  | 1.23br |
| C9 | 22.00 | 1.24 (m) |  | 1.23br |
| C10 | 13.87 | 0.90 – 0.76(m) |  |  |

a, ^1^H and ^13^C NMR Chemical Shift was summarized from Lindum *et al* [22]. b, in this study, the two ^13^C NMR chemical shift of Leu and Ile were different with Lindum *et al* [22].
