# Supplementary material for: Analysis of the genomic sequences and metabolites of Serratia surfactantfaciens sp. nov. YD25T that simultaneously produces prodigiosin and serrawettin W2
Source: BMC Genomics. 2016 Nov 3;17:865. doi: 10.1186/s12864-016-3171-7 (PMC5094094; doi:10.1186/s12864-016-3171-7)
Supplement: Additional file 5: Table S2. — Deduced functions and homologues of the gene products from the pig clusters. The relatedness of each YD25T pig protein to its homologue in S. marcescens ATCC 274, S. plymuthica AS 13, and Serratia sp. ATCC 39006 is shown in the comparison of Serratia pig protein percentage sequence identity column. (DOCX 15 kb) [file 12864_2016_3171_MOESM5_ESM.docx]

**Table S2.** **Deduced functions and homologues of the gene products from the *pig* clusters.** The relatedness of each YD25^T^ pig protein to its homologue in *S. marcescens* ATCC 274, *S. plymuthica* AS 13, and *Serratia* sp. ATCC 39006 is shown in the comparison of *Serratia* pig protein percentage sequence identity column.

| Protein | Comparison of Pig protein (aa / Identity) | | | | | | | Putative Function |
| --- | --- | --- | --- | --- | --- | --- | --- | --- |
|  | *Serratia* strainYD25^T^ | *Serratia marcescens*  ATCC 274 | | *Serratia plymuthica*  AS 13 | | *Serratia* sp.  ATCC 39006 | |  |
| PigA | 385 | 385 | 374/385 (97 %) | 386 | 307/385 (80 %) | 386 | 303/385 (79 %) | anyl-CoA dehydrogenase |
| PigB | 669 | 671 | 646/671 (96 %) | 676 | 477/650 (73 %) | 670 | 476/659 (72 %) | no assigned function |
| PigC | 888 | 888 | 880/888 (99 %) | 890 | 703/888 (79 %) | 890 | 665/885 (75 %) | phosphortransferase |
| PigD | 903 | 904 | 883/904 (98 %) | 866 | 760/867 (88 %) | 866 | 736/867 (85 %) | no assigned function |
| PigE | 853 | 853 | 847/853 (99 %) | 853 | 763/853 (89 %) | 853 | 729/853 (85 %) | aminotransferase |
| PigF | 338 | 338 | 337/338 (99 %) | 341 | 284/338 (84 %) | 348 | 279/338 (83 %) | O-methyltransferase |
| PigG | 87 | 87 | 87/87 (100 %) | 87 | 70/87 (80 %) | 87 | 68/87 (78 %) | peptidyl carrier protein |
| PigH | 648 | 648 | 639/647 (99 %) | 656 | 526/656 (80 %) | 653 | 512/654 (78 %) | aminotransferase |
| PigI | 491 | 490 | 475/491 (97 %) | 489 | 338/491 (69 %) | 491 | 326/483 (67 %) | L-prolyl-AMP ligase |
| PigJ | 762 | 762 | 749/762 (98 %) | 770 | 538/768 (70 %) | 770 | 520/768 (68 %) | β-ketomyristol-ACP synthase |
| PigK | 104 | 104 | 104/104 (100 %) | 104 | 79/103 (77 %) | 104 | 74/102 (73 %) | no assigned function |
| PigL | 232 | 215 | 202/215 (94 %) | 214 | 97/172 (56 %) | 234 | 104/174 (60 %) | 4'-phosphopantetheinyltransferase |
| PigM | 352 | 352 | 347/352 (99 %) | 359 | 221/345 (64 %) | 359 | 212/343 (62 %) | no assigned function |
| PigN | 242 | 242 | 237/242 (98 %) | 242 | 190/242 (79 %) | 242 | 184/242 (76 %) | oxidoreductase |
| PigO | 0 | 0 |  | 0 |  | 157 |  | no assigned function |
