# Supplementary material for: Analysis of the genomic sequences and metabolites of Serratia surfactantfaciens sp. nov. YD25T that simultaneously produces prodigiosin and serrawettin W2
Source: BMC Genomics. 2016 Nov 3;17:865. doi: 10.1186/s12864-016-3171-7 (PMC5094094; doi:10.1186/s12864-016-3171-7)
Supplement: Additional file 6: Table S3. — Deduced functions and homologues of the gene products from the lsr clusters. The relatedness of each YD25T Lsr protein to its homologue in Salmonella typhimurium LT2, E. coli K-12, and E. fergusonii ATCC 35469 is shown in the comparison of Lsr protein percentage sequence identity column. (DOCX 15 kb) [file 12864_2016_3171_MOESM6_ESM.docx]

**Table S3. Deduced functions and homologues of the gene products from the *lsr* clusters.** The relatedness of each YD25^T^ Lsr protein to its homologue in *Salmonella typhimurium* LT2, *E. coli* K-12, and *E. fergusonii* ATCC 35469 is shown in the comparison of Lsr protein percentage sequence identity column.

| Protein | Comparison of Lsr protein (aa / Identity) | | | | | | | Putative Function |
| --- | --- | --- | --- | --- | --- | --- | --- | --- |
|  | *Serratia* strainYD25^T^ | *Sal. typhimurium*  LT2 | | *E. coli*  K-12 | | *E. fergusonii*  ATCC 35469 | |  |
| LsrA | 505 | 511 | 173/497 (35 %) | 511 | 176/502 (35 %) | 511 | 169/489 (35 %) | sugar ABC transporter ATP-binding protein |
| LsrB | 327 | 340 | 79/312 (25 %) | 340 | 78/312 (25 %) | 340 | 71/287 (25 %) | ABC transporter substrate-binding protein |
| LsrC | 328 | 347 | 84/312 (27 %) | 342 | 83/283 (29 %) | 342 | 90/309 (29 %) | sugar ABC transporter permease |
| LsrD | 344 | 333 | 93/348 (27 %) | 330 | 93/338 (28 %) | 330 | 63/205 (31 %) | sugar ABC transporter permease |
| LsrE | 212 | 254 | 61/212 (29 %) | 0 | - | 0 | - | allulose-6-phosphate 3-epimerase |
| LsrF | 302 | 291 | 10/36 (28 %) | 291 | 10/42 (24 %) | 291 | 11/39 (28 %) | autoinducer-2 aldolase  utoinducer-2 (AI-2) aldolase |
| LsrG | 97 | 97 | 71/96 (74 %) | 96 | 71/96 (74 %) | 96 | 71/96 (74 %) | autoinducer-2 modifying protein |
| LsrK | 521 | 530 | 408/518 (79 %) | 530 | 410/519 (79 %) | 530 | 405/519 (78 %) | [autoinducer-2 kinase](http://blast.ncbi.nlm.nih.gov/Blast.cgi#alnHdr_518283699) |
| LsrR | 320 | 319 | 87/307 (28 %) | 317 | 83/309 (27 %) | 317 | 84/309 (27 %) | transcriptional regulator |
| LuxS | 171 | 171 | 148/171 (87 %) | 171 | 146/171 (85 %) | 171 | 123/147 (84 %) | [S-ribosylhomocysteinase](http://blast.ncbi.nlm.nih.gov/Blast.cgi#alnHdr_518284925) |
