# Supplementary material for: Analysis of the genomic sequences and metabolites of Serratia surfactantfaciens sp. nov. YD25T that simultaneously produces prodigiosin and serrawettin W2
Source: BMC Genomics. 2016 Nov 3;17:865. doi: 10.1186/s12864-016-3171-7 (PMC5094094; doi:10.1186/s12864-016-3171-7)
Supplement: Additional file 8: Table S4. — The less than 98.7 % 16S rRNA gene sequence similarities between YD25T and type strains of phylogenetically related species. (DOCX 12 kb) [file 12864_2016_3171_MOESM8_ESM.docx]

**Table S4. The less than 98.7 % 16S rRNA gene sequence similarities between YD25^T^ and type strains of phylogenetically related species.**

| Species | 16S rRNA gene sequence similarity (%) with YD25^T^ |
| --- | --- |
| *Serratia ficaria* | 98.3 |
| *Serratia ureilytica* | 98.2 |
| *Serratia odorifera* | 98.1 |
| *Serratia entomophila* | 97.8 |
| *Serratia rubidaea* | 97.6 |
| *Serratia vespertilionis*  *Serratia plymuthica*  *Serratia symbiotica*,  *Serratia grimesii*,  *Serratia liquefaciens*,  *Serratia quinivorans*,  *Serratia glossinae*,  *Serratia proteamaculans*  *Serratia myotis*  *Serratia fonticola*. | 97.6  97.3  97.2  96.9  96.9  96.9  96.7  96.7  96.6  96.5 |
