# Supplementary material for: Analysis of the genomic sequences and metabolites of Serratia surfactantfaciens sp. nov. YD25T that simultaneously produces prodigiosin and serrawettin W2
Source: BMC Genomics. 2016 Nov 3;17:865. doi: 10.1186/s12864-016-3171-7 (PMC5094094; doi:10.1186/s12864-016-3171-7)
Supplement: Additional file 10: Table S5. — 16S rRNA, rpoB, atpD, gyrB and infB gene sequence similarities between YD25T and type strains of phylogenetically related species. (DOCX 13 kb) [file 12864_2016_3171_MOESM10_ESM.docx]

**Table S5. 16S rRNA, *rpo*B, *atp*D, *gyr*B and *inf*B gene sequence similarities between YD25^T^ and type strains of phylogenetically related species.**

| Species | Gene sequence similarity (%) with YD25^T^ | | | | | |
| --- | --- | --- | --- | --- | --- | --- |
|  | 16S rRNA | *rpo*B | *atp*D | *gyr*B | *inf*B | * |
| *Serratia nematodiphila* | 99.7 | 98.7 | 98.8 | 97.3 | 97.0 | 98.0 |
| *Serratia marcescens* | 99.4 | 98.7 | 98.6 | 96.8 | 97.2 | 98.0 |
| *Serratia ficaria* | 98.3 | 97.0 | 96.0 | 93.6 | 93.6 | 95.0 |
| *Serratia ureilytica* | 98.2 | 98.9 | 99.1 | 97.5 | 96.4 | 98.0 |
| *Serratia odorifera* | 98.1 | 95.6 | 92.8 | 88.5 | 91.1 | 92.0 |
| *Serratia entomophila* | 97.8 | 96.7 | 96.0 | 93.0 | 92.8 | 94.6 |
| *Serratia rubidaea* | 97.6 | 95.4 | 93.3 | 88.1 | 89.2 | 91.6 |
| *Serratia plymuthica* | 97.3 | 94.3 | 94.1 | 89.6 | 89.7 | 92.0 |

*concatenated partial *rpo*B, *atp*D, *gyr*B and *inf*B.
