# Supplementary material for: Analysis of the genomic sequences and metabolites of Serratia surfactantfaciens sp. nov. YD25T that simultaneously produces prodigiosin and serrawettin W2
Source: BMC Genomics. 2016 Nov 3;17:865. doi: 10.1186/s12864-016-3171-7 (PMC5094094; doi:10.1186/s12864-016-3171-7)
Supplement: Additional file 12: — Description of Serratia surfactantfaciens sp. nov. (DOCX 16 kb) [file 12864_2016_3171_MOESM12_ESM.docx]

**Description of *Serratia surfactantfaciens* sp. nov.**

*Serratia surfactantfaciens* (sur.fact.ant.faci’ens. N.L. part. adj. One of the secondary metabolites produced by the strain is a biosurfactant).

Cells are Gram-negative, motile, non-spore-forming short rods, 1.2~1.4×0.6~0.7 μm and with one week flagellum. Colonies on a KB agar are red and branching rough. Grow at 10-42 °C, optimally at a temperature of 28-30 °C, pH 2.0-11.0, with optimum growth at pH 5.0-9.0, and tolerant to 8 % (w/v) NaCl in nutrient broth. Catalase is positive and oxidase is negative. Phosphatase, ornithine decarboxylase, lysine decarboxylase, L-pyrrolydonyl arylamidase, L-proline arylamidase, glu-gly-arg arylamidase, β-glucosidase, and β-N-acetyl glucosaminidase activities are present, but α-glucosidase, β-galactosidase, phenylalanine decarboxylase, ala-phe-pro arylamidase, glutamyl arylamidase, β-alanine arylamidase, glycine arylamidase, tyrosine arylamidase, γ-glutamyl transferase, β-xylosidase, lipase or β-glucoronidase activities are absent. Negative for H_2_S production, but methyl red test, Voges-Proskauer reaction, fermentation/glucose, L-lactate alkalinisation, succinate alkalinisation and O/129 resistance (comp.vibrio.) are positive. Tween 80, amygdalin and gelatin are hydrolysed. Utilizes α-D-glucose, D-glucose-6-phosphate, 3-methyl glucose, sucrose, D-mannose, dextrin, D-cellobiose, gentiobiose, α-D-lactose, D-melibiose, D-fructose, D-fucose, L-fucose, D-fructose-6-phosphate, D-trehalose, D-turanose, D-maltose, D-galactose, D-mannitol, adonitol, D-arabitol, glycerol, L-arabinitol, D-sorbitol, cumaric acid, D-serine, L-serine, glycyl-L-proline, L-alanine, L-aspartic acid, L-glutamic acid, L-histidine, L-pyroglutamic acid, p-hydroxy-phenylacetic acid, L-lactic acid, citric acid, α-keto-glutaric acid, L-malic acid, acetic acid, formic acid, D-gluconic acid, D-glucuronic acid, fusidic acid, citrate (sodium), 5-keto-D-gluconate, D-galacturonic acid lactone, inosine, myo-inositol, 1 % sodium lactate, N-acetyl-D-glucosamine, N-acetyl-β-D-mannosamine, N-acetyl-D-galactosamine, guanidine, sodium myristyl sulfate, tetrazolium violet, and tetrazolium blue, but does not utilize arabinose, stachyose, palatinose, L-rhamnose, D-tagatose, D-raffinose, D-cellobiose, D-salicin, malonate, sodium bromate, L-malate, L-lactate, L-histidine, D-malic acid, nalidixic acid, γ-amino-butryric acid, β-hydroxy-D-butyric acid, propionic acid or D-lactic acid methyl ester. The major fatty acids are C_16:0_, summed feature 3 (C_16:1_ *ω7c*/C_16:1_ *ω6c*), summed feature 8 (C_18:1_ *ω7c*/C_18:1_ *ω6c*), C_17:0_ cyclo, summed feature 2 (C_14:0_ 3-OH/C_16:1_ iso), C_14:0_, C_14:0_ 2-OH, C_12:0_ 2-OH, C_12:0_, C_19:0_ cyclo *ω8c* and C_18:0_. YD25^T^ is resistant to ampicillin, tetracycline, carbenicillin, vancomycin, troleandomycin, rifamycin SV and lincomycin, but sensitive to gentamicin, streptomycin, kanamycin, apramycin and chloramphenicol. The DNA G+C content of the type strain genomic DNA is 59.62 mol %. A total length of genome is approximately 5,115,690 bp.

The type strain is strain *S. surfactantfaciens* YD25^T^ (=CCTCC AB 2015384; =KCTC 42987), isolated from rhizosphere soils under continuously planting burley tobacco in Fujian Province, China.
